# Modular Stereoselective Synthesis of Sex Pheromone of *Lambdina fiscellaria lugubrosa* (Hulst) and Discovery of Cross-Species Attraction in *Semiothisa cinerearia* (Bremer & Grey)

**DOI:** 10.3390/molecules30214216

**Published:** 2025-10-28

**Authors:** Yun Zhou, Jionglin Wang, Yueru Zhang, Xiaochen Fu, Xiaoyang Li, Jianan Wang, Xianchang Wang, Jianhua Zhang, Yanbing Gu, Jinlong Han, Jiangchun Zhong, Chenggang Shan

**Affiliations:** 1 Institute of Industrial Crops, Shandong Academy of Agricultural Sciences, Jinan 250100, China; zysass2021@163.com (Y.Z.); archangw@163.com (X.W.); zhangjianhua198904@163.com (J.Z.); goldendragonh@163.com (J.H.); 2 Department of Applied Chemistry, China Agricultural University, Beijing 100193, China; s20223102112@cau.edu.cn (J.W.); b20233100844@cau.edu.cn (X.L.); xxjnwang@163.com (J.W.); 3 School of Pharmacy, Shandong University of Traditional Chinese Medicine, Jinan 250355, China; 13553169667@163.com (Y.Z.); fxc13345203071@163.com (X.F.); 4 Sishui County Agricultural Technology Extension Center, Sishui 273200, China; guyanbing2010@163.com

**Keywords:** stereoselective synthesis, sex pheromone, western hemlock looper, cross-species attraction

## Abstract

The western hemlock looper, *Lambdina fiscellaria lugubrosa* (Hulst) is a destructive defoliator of coniferous forests and a major cause of economic losses in forestry. A novel and efficient stereoselective synthesis of the sex pheromone of the western hemlock looper (**1**, **2** and **3**) has been successfully achieved. The synthetic strategy integrates several key transformations, including Evans’ chiral auxiliaries, Grignard cross-coupling, hydroboration–oxidation, sulfone alkylation, and hydrogenation, providing an efficient and scalable approach for sex pheromone production. The three synthesized pheromone components were subsequently tested for their ability to attract *Semiothisa cinerearia* (Bremer & Grey) using both Y-tube and cage bioassays. Notably, compound **1** exhibited a cross-species attractive effect on *S. cinerearia*, a species that had not previously been documented to respond to the pheromone of *L. fiscellaria lugubrosa*. This discovery underscores the potential for cross-species attraction, broadens our understanding of pheromone specificity, and emphasizes the value of stereoselectively synthesized pheromones as molecular tools for cross-species pest monitoring and integrated pest management.

## 1. Introduction

The western hemlock looper, *L. fiscellaria lugubrosa*, is a member of the Geometridae family and a significant defoliator of its primary host, the western hemlock (*Tsuga heterophylla*), as well as associated conifer species in North America and Canada [1,2,3,4]. Its larvae feed on various conifer species associated with hemlock, including Sitka spruce (*Picea sitchensis*), subalpine fir (*Abies lasiocarpa*), grand fir (*Abies grandis*), Pacific silver fir (*Abies amabilis*), western white pine (*Pinus monticola*), and western redcedar (*Thuja plicata*), among others [5,6]. These inchworm-like caterpillars consume foliage of all ages, and severe defoliation during outbreaks can lead to tree mortality after just one year of feeding [3,7]. Therefore, monitoring and controlling this pest to prevent its outbreak is especially important.

**Figure 1:**
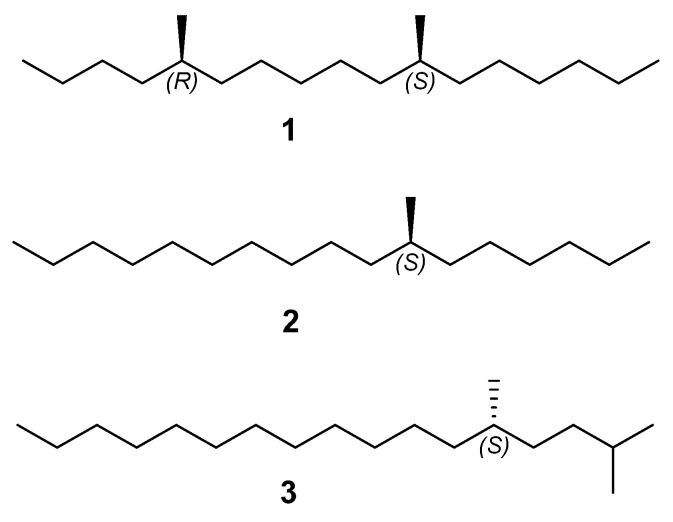
The sex pheromone components of *L. fiscellaria lugubrosa*.

Pheromone-based pest management is integral to sustainable agriculture and forestry, offering targeted, eco-friendly alternatives to chemical pesticides [8]. Strategies such as mating disruption, mass trapping, and monitoring exploit insect communication pathways to suppress pest populations while minimizing non-target impacts and environmental contamination. Additionally, these methods mitigate the risk of pesticide resistance, enhancing long-term control efficacy [9]. In, 1993, Slessor and coworkers first recognized (5*R*,11*S*)-5,11-dimethylheptadecane (**1**), (*S*)-7-methylheptadecane (**2**) and (*S*)-2,5-dimethylheptadecane (**3**) as the sex pheromone of the western hemlock looper by means of field trapping experiments (Figure 1) [10,11,12]. Given its significant role in the monitoring and control of this pest, there have been several reports on syntheses of this pheromone components based on chiral pool strategy. Slessor’s group firstly reported the synthesis of the sex pheromone of the western hemlock looper from methoxybornyl sulfoxides as a chiral precursor and Bakers’ yeast reduction [10,11]. In 1996, Mori’s group realized the synthesis of (5*R*,11*S*)-5,11-dimethylheptadecane (**1**) in 13 steps from the enantiomers of methyl3-hydroxy-2-methylpropanoate, and 2,5-dimethylheptadecane (**3**) with a total yield of 29% in 5 steps from (*S*)-citronellol [13]. In 2023, Zhong’s group achieved the synthesis of (5*R*,11*S*)-5,11-dimethylheptadecane (**1**) and (*S*)-7-methylheptadecane (**2**) using the enantiomers of 2-methyloxiraneoxide as chiral sources [14]. While the previously reported routes were efficient, our study focuses on developing a more diverse and convergent synthetic strategy, enabling flexible assembly of structurally related pheromone analogues from shared chiral building blocks. To facilitate the utilization of the sex pheromone, the development of a novel and efficient synthetic route based on a chiral auxiliary strategy is of great significance.

While pheromones are typically considered species-specific, increasing evidence suggests that pheromone components may also elicit responses in non-target species. Such cross-attraction phenomena are of particular interest in chemical ecology, as they can reveal evolutionary relationships in pheromone communication systems and offer potential for developing broad-spectrum monitoring tools [15,16,17]. In this study, we report the stereoselective synthesis of three sex pheromone components of *L. fiscellaria lugubrosa* and evaluate their behavioral activity in *S. cinerearia*, another geometrid species of ecological relevance. Laboratory-based Y-tube olfactometer and cage bioassays were employed to assess the potential of these compounds to act as attractants. Interestingly, component **1** was found to elicit cross-attractive effect on *S. cinerearia*, representing the first evidence of interspecies pheromone communication involving *L. fiscellaria lugubrosa*. Our work presents two complementary advances that have not been simultaneously achieved in previous studies. First, we developed a modular and stereoselective synthetic strategy based on Evans’ chiral auxiliaries, which offers a shorter, more convergent, and scalable route compared with previously reported methods employing chiral building blocks or long linear sequences. Second, we demonstrate a novel biological phenomenon in which one of the synthesized pheromone components functions as a cross-attractant in *S. cinerearia*, as supported by both Y-tube olfactometer and cage bioassays. These results not only expand current understanding of pheromone-mediated communication in geometrid moths but also provide a new chemical–ecological basis for developing sustainable pest management strategies that harness cross-attraction mechanisms.

## 2. Results

### 2.1. Synthesis of Sex Pheromone of L. fiscellaria lugubrosa ***1*** and ***2***

**Scheme 1:**
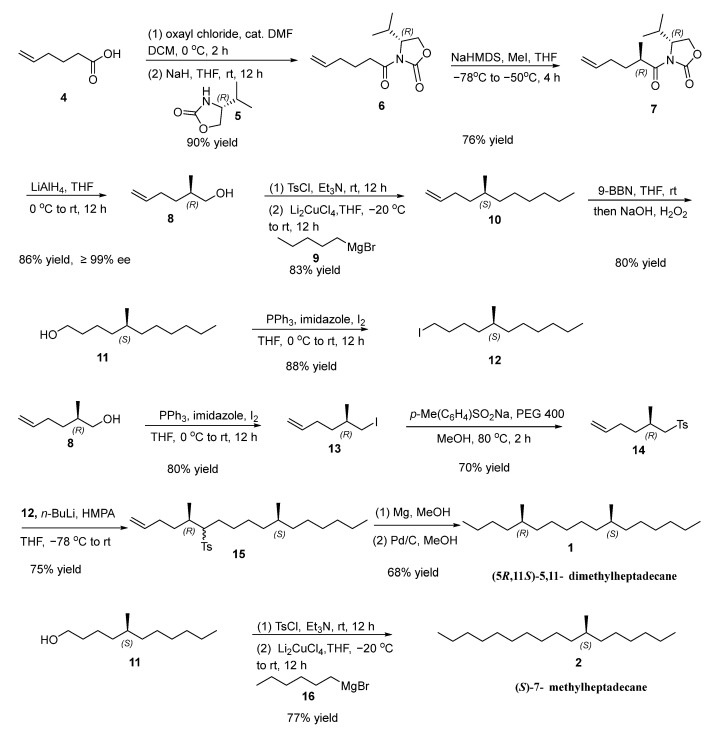
Synthesis of (5*R*,11*S*)-5,11-dimethylheptadecane (**1**) and (*S*)-7-methylheptadecane (**2**).

The synthesis of sex pheromone of *L. fiscellaria lugubrosa* **1** and **2** is summarized in Scheme 1. Treatment of hex-5-enoic acid (**4**) with oxalyl chloride produced the corresponding acyl chloride, which was then acylated with (*R*)-4-isopropyloxazolidin-2-one (**5**) to yield (*R*)-3-(hex-5-enoyl)-4-isopropyloxazolidin-2-one (**6**) in 90% yield [18]. Subsequent diastereoselective methylation and reductive cleavage resulted in allylic alcohol **8**, exhibiting an enantiomeric excess (ee) of 99% as estimated by ^1^H NMR analysis of its Mosher ester [19]. Its specific rotation {[α]_D_^25^ = +7.17 (c 0.61, CHCl_3_)} was consistent with the literature value {[α]_D_^20^ = +9.1 (c 3.03, CHCl_3_)} for (*R*)-2-methylhex-5-en-1-ol (**8**), confirming that compound **8** possesses the R configuration [20,21]. The tosylate of **8** was then coupled with pentylmagnesium bromide (**9**) under the catalysis of lithium tetrachlorocuprate (II) (Li_2_CuCl_4_), affording (*S*)-5-methylundec-1-ene (**10**) in 83% yield [22,23]. Hydroboration–oxidation of **10** using 9-borabicyclo [3.3.1]nonane (9-BBN) and hydrogen peroxide (H_2_O_2_) resulted in (*S*)-5-methylundecan-1-ol (**11**) with an 80% yield [24]. Subsequent iodination with I_2_/PPh_3_/imidazole in THF furnished chiral alkyl iodide **12** in 88% yield [25]. Parallel iodination of (*R*)-2-methylhex-5-en-1-ol (**8**) afforded(*R*)-6-iodo-5-methylhex-1-ene (**13**) in 80% yield [25], which was transformed into sulfone **14** in 70% yield [26]. Sulfone **14** was then alkylated with chiral alkyl iodide **12** to give **15** with a 5,11-dimethyl skeleton by using *n*-butyllithium and hexamethylphosphoric triamide in 75% yield [26,27]. Reductive desulfonylation of **15** with magnesium in methanol, followed by Pd/C-catalyzed hydrogenation, delivered (5*R*,11*S*)-5,11-dimethylheptadecane (**1**) in 68% yield [26,28]. The specific rotation of **1** was −0.93 (c 0.63, CHCl_3_), aligning closely with the reported value in the literature of −0.90 (c 1.99, Hexane). Additionally, the structures of **1** was confirmed using ^1^H NMR, ^13^C NMR and HRMS spectra, consistent with the literature reports [13].

In parallel, alcohol **11** was converted to its tosylate, which coupled with hexylmagnesium bromide under Li_2_CuCl_4_ catalysis to furnish (*S*)-7-methylheptadecane (**2**) in 77% yield [22]. The sex pheromone of *L. fiscellaria lugubrosa* **2** was characterized with ^1^H NMR, ^13^C NMR (Supplementary Materials) and HRMS spectra. The specific rotation of **2** was −1.76 (c 0.87, CHCl_3_), which aligns closely with the published literature value of −1.97 (c 3.04, CHCl_3_) [14].

### 2.2. Synthesis of Sex Pheromone of L. fiscellaria lugubrosa ***3***

**Scheme 2:**
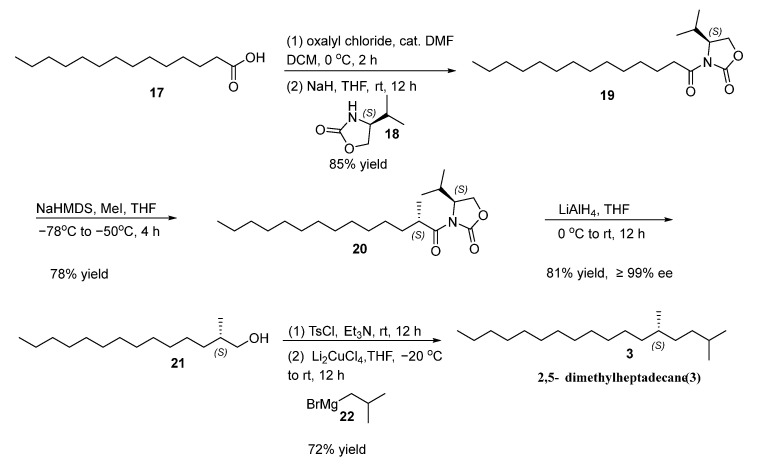
Synthesis of (*S*)-2,5-dimethylheptadecane (**3**).

The sex pheromone of *L. fiscellaria lugubrosa* **3** was prepared according to the similar strategy of constructing **1**, as shown in Scheme 2. Starting from tetradecanoic acid, we synthesized (*S*)-4-isopropyl-3-tetradecanoyloxazolidin-2-one (**19**) using oxalyl chloride and (*S*)-4-isopropyloxazolidin-2-one in 85% yield [18]. Subsequent diastereoselective methylation with iodomethane (MeI) in the presence of sodium bis(trimethylsilyl)amide (NaHMDS) produced (*S*)-4-isopropyl-3-((*S*)-2-methyltetradecanoyl)oxazolidin-2-one (**20**) in 78% yield [19]. Reductive cleavage of the chiral auxiliary with Lithium aluminum hydride (LiAlH_4_) gave (*S*)-2-methyltetradecan-1-ol (**21**) in 81% yield and 99% enantiomeric excess (ee), as estimated by ^1^H NMR spectra of its Mosher ester. The specific rotation of **21** {[α]_D_^20^ = −10.60 (c 1.10, CHCl_3_)} closely matched the literature value {[α]_D_^20^ =−11.36 (c 0.88, CHCl_3_)}, allowing for the assignment of its absolute configuration as S [29,30]. The final transformation into the target sex pheromone **3** was achieved through the tosylation of compound **21** with TsCl, followed by coupling reaction with *iso*-butylmagnesium bromide in a yield of 72% [22,23]. The structure of **3** was characterized by ^1^H NMR, ^13^C NMR, HRMS and specific rotation, all of which were consistent with the literature reference [13].

### 2.3. Evaluation of the Cross-Species Attractive Effect on Male S. cinerearia

**Figure 2:**
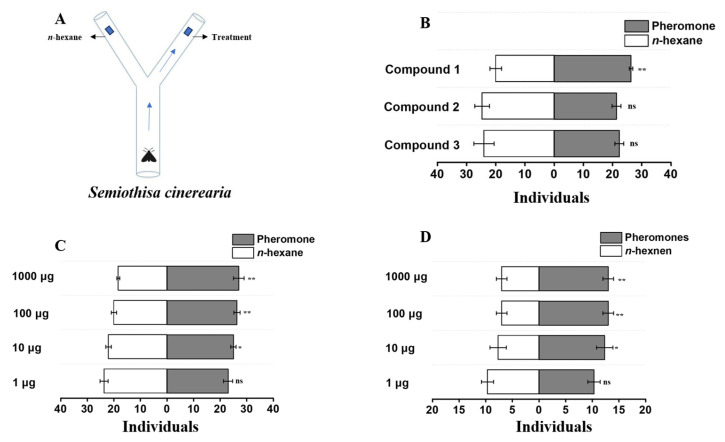
Evaluation of the cross-species attractive effect. (**A**) Schematic of the Y-tube olfactometer for male *S. cinerearia* behavioral bioassays. (**B**) Y-Tube olfactometer assay of male *S. cinerearia* behavioral responses to 0.1 mg of compound **1**,**2** and **3** (*n* = 3 replicates per compound). (**C**) Y-Tube olfactometer assay of male *S. cinerearia* behavioral responses to different doses of compound **1** (*n* = 3 replicates per dose). (**D**) Cage bioassay for evaluating male *S. cinerearia* behavioral responses to different doses of compound 1 (*n* = 3 replicates per dose). Statistically significant differences were evaluated using Student’s *t*-test (*, *p* < 0.05; **, *p* < 0.01; ns, no significant difference).

Building upon our efficient asymmetric syntheses and stereochemical assignments of the three *L. fiscellaria lugubrosa* pheromone components (**1**–**3**), we next examined their ecological activity in a non-conspecific geometrid. Given the potential conservation of pheromone biosynthesis and recognition within Geometridae, we selected *S. cinerearia* as a model to evaluate cross-species attraction [31]. *S. cinerearia* similar to *L. fiscellaria lugubrosa*, belongs to Geometridae, which is one of the most species-rich families of lepidopteran insects. It also ranks among the most economically significant pests affecting the Chinese scholar tree [32]. We conducted Y-tube olfactometer and cage bioassay to determine the activity and dose–response profiles of compounds **1**–**3** (Figure 2) [33,34,35], thereby testing the hypothesis of cross-species recognition and informing broader pheromone-based applications.

We first employed a Y-tube olfactometer (Figure 2A) to evaluate the behavioral responses of male *S. cinerearia* to the three pheromone components (**1**, **2**, and **3**) of *L. fiscellaria lugubrosa*, using 0.1 mg as the test dose. As shown in Figure 2B, compound **1** elicited both attraction and retention effects in males, whereas compounds **2** and **3** did not induce behavioral responses. To further examine the dose-dependent preference, we tested compound **1** at four concentrations (1 μg, 10 μg, 100 μg, and 1000 μg). The results (Figure 2C) demonstrated that compound **1** began to exhibit detectable attractiveness to males at doses above 10 μg. To validate these findings under more realistic conditions, we conducted cage bioassays with the same four doses of compound **1**. The results were consistent with those from the Y-tube assays and showed an obvious dose-dependent effect; attraction was observed at ≥10 μg, while a good cross-attractive effect was recorded at doses ≥100 μg (Figure 2D). These results reveal that sex pheromones may retain conserved recognition mechanisms across species. Compound **1** plays a pivotal role not only in intraspecific communication of *L. fiscellaria lugubrosa* but also triggers cross-attraction in *S. cinerearia*. This expands our understanding of pheromone specificity and evolutionary commonality, while providing a novel molecular tool for cross-species pest monitoring.

## 3. Discussion

In this study, we successfully developed a novel and efficient stereoselective synthesis of the three sex pheromone components of the western hemlock looper, *L. fiscellaria lugubrosa*. Starting from acid **4**, (5*R*,11*S*)-5,11-dimethylheptadecane (**1**) and (*S*)-7-methylheptadecane (**2**) were synthesized in a total of 8 and 6 steps, respectively, with overall yields of 18% and 30%. The overall yield of (*S*)-2,5-dimethylheptadecane (**3**) was 39%, achieved from acid **17** in 4 steps. All products exhibited optical purities greater than 99%.

Compared to reported synthetic methods, our approach offers advantages such as inexpensive starting materials, a shorter synthetic route, good overall yields, and excellent optical purity of the target compounds. Slessor’s biocatalytic asymmetric synthesis of (*S*)-2,5-dimethylheptadecane (**3**) involved eight steps with an overall yield of only 13%, making this approach impractical for large-scale production [10,11]. Mori’s classical synthesis of (5*R*,11*S*)-5,11-dimethylheptadecane (**1**) required 19 consecutive steps, which severely limits its scalability [13]. Zhong’s route employed chiral 2-methyloxirane as the chiral source to synthesize (5*R*,11*S*)-5,11-dimethylheptadecane (**1**) in 13 steps with an overall yield of 17%. Although the chiral starting materials used in Zhong’s strategy are inexpensive and readily available, the reaction conditions are relatively demanding, often requiring elevated temperatures, and the optical purity of the final product reached only 96%, which makes it difficult to scale up for practical application [14]. In contrast, our synthetic strategy was deliberately designed with scalability, efficiency, and practicality in mind. The use of Evans’ chiral auxiliaries provides a robust and easily removable stereochemical control element, while the resulting intermediates are stable and suitable for multigram-scale synthesis. By employing inexpensive starting carboxylic acids and well-established transformations—such as hydroboration–oxidation, sulfone alkylation, and Grignard cross-coupling—the present route circumvents the need for multiple protecting-group manipulations in previously reported methods. Importantly, the integration of Evans’ auxiliary chemistry with a convergent Grignard cross-coupling and sulfone alkylation strategy for the assembly of methyl-branched long-chain alkanes—the characteristic structural motif of geometrid pheromones—has not, to our knowledge, been previously reported in a concise and high-optical-purity synthetic route. Our approach offers several distinct advantages: (i) exceptional stereochemical control with optical purities exceeding 99%, (ii) a reduced number of protecting-group steps and shorter linear sequences for key intermediates, and (iii) modularity that facilitates straightforward analogue synthesis with varied chain lengths or branching patterns without the need to redesign the entire route. Overall, these methodological advances enhance the practical accessibility of pheromone components and provide a versatile platform for structure–activity relationship exploration and scalable production, thereby supporting future applications in integrated pest management (IPM).

Beyond the achievement in asymmetric synthesis, the behavioral assays revealed a biologically important and unexpected finding. Among the three synthesized pheromone components, compound **1** exhibited cross-attractive effect on male *S. cinerearia*, a geometrid moth not previously reported to respond to *L. fiscellaria lugubrosa* sex pheromone. Y-tube olfactometer tests demonstrated that compound **1** elicited a dose-dependent behavioral response, with attraction detectable at 10 μg and increasing significantly at ≥100 μg. These results were further validated under more naturalistic conditions using cage bioassays, which confirmed that compound **1** consistently attracted male *S. cinerearia* at higher doses. While *S. cinerearia* and *L. fiscellaria lugubrosa* belong to different genera, both are Geometrid moths, which may explain partial overlap in pheromone communication systems. In the future, we will continue to investigate the interaction mechanism between compound **1** and the olfactory receptors of *S. cinerearia* to further elucidate the biological basis of this phenomenon. Our finding suggests that structurally similar pheromone components may act as interspecific cues, potentially influencing mating behaviors or serving as ecological signals across species boundaries.

## 4. Materials and Methods

### 4.1. General Information for Synthesizing Sex Pheromone of L. fiscellaria lugubrosa

All reactions were performed under an inert atmosphere of argon, utilizing a Schlenk line system. Reagents were sourced commercially and used without further purification, while solvents underwent distillation before application, following standard protocols. Proton NMR (^1^H NMR) spectra were obtained at 500 MHz using TMS at δ 0.00 ppm or CDCl_3_ at δ 7.26 ppm, and carbon NMR (^13^C NMR) spectra were recorded at 126 MHz, with CDCl_3_ set at δ 77.16 ppm as the internal standard, employing a Bruker DP-X500 spectrometer (Bruker Corporation, Beijing, China). High-resolution mass spectrometry (HRMS) data were collected using a Waters LCT Premier™ system (Waters Corporation, Beijing, China) equipped with an electrospray ionization (ESI) source. Optical rotation measurements were taken with a Rudolph Research Analytical AUTOPOL-IV polarimeter (Rudolph Research Analytical, Beijing, China).

*Synthesis of (R)-3-(hex-5-enoyl)-4-isopropyloxazolidin-2-one* (**6**)

Hex-5-enoic acid (**4**) (1.72 g, 15 mmol) was dissolved in dry dichloromethane (DCM) (20 mL) at 0 °C under an argon atmosphere. Oxalyl chloride (2.86 g, 22.5 mmol) and a catalytic amount of DMF were then added slowly; the reaction mixture was stirred for 2 h at 0 °C. The solvent was removed under reduced pressure to give the crude hex-5-enoyl chloride as a slight yellow oil.

NaH (0.9 g, 60% in mineral oil, 22.5 mmol) was dissolved in dry tetrahydrofuran (20 mL) at 0 °C under an argon atmosphere. (*R*)-4-isopropyloxazolidin-2-one (**5**) (3.46 g, 19.5 mmol) in tetrahydrofuran (20 mL) was added slowly. The resulting mixture was warmed to 25 °C and stirred for 2 h. Then, the crude hex-5-enoyl chloride in dry tetrahydrofuran (10 mL) was added slowly at 0 °C, the resulting mixture was allowed to rise to room temperature and stirred for another 4 h. The reaction was quenched with saturated aqueous NH_4_Cl solution (20 mL). The layers were separated, and the aqueous phase was extracted with ethyl acetate (3 × 50 mL). The combined organic layers were washed with brine (50 mL) and dried over anhydrous Na_2_SO_4_, concentrated under reduced pressure. The residue was purified by silica gel column chromatography (ethyl acetate/petroleum ether = 1:5) to provide (*R*)-3-(hex-5-enoyl)-4-isopropyloxazolidin-2-one (**6**) as a light yellow oil (3.04 g, 90% yield). [α]_D_^25^ = −67.66 (c 1.80, CHCl_3_). ^1^H NMR (400 MHz, Chloroform-*d*) δ 5.85–5.75 (m, 1H), 5.12–4.94 (m, 2H), 4.45–4.41 (m 1H), 4.30–4.17 (m, 2H), 3.03–2.84 (m, 2H), 2.41–2.33 (m, 1H), 2.13 (q, *J* = 7.2 Hz, 2H), 1.83–1.71 (m, 2H), 0.91 (d, *J* = 7.0 Hz, 3H), 0.87 (d, *J* = 6.9 Hz, 3H). ^13^C NMR (101 MHz, CDCl_3_) δ 173.20, 154.14, 137.91, 115.36, 63.42, 58.45, 34.90, 33.11, 28.46, 23.63, 18.04, 14.73. HRMS (ESI, *m*/*z*): calculated for [M + Na]^+^ C_12_H_19_NO_3_Na 248.1257, found: 248.1240.

*Synthesis of (R)-4-isopropyl-3-((R)-2-methylhex-5-enoyl)oxazolidin-2-one* (**7**)

(*R*)-3-(hex-5-enoyl)-4-isopropyloxazolidin-2-one (**6**) (3.38 g, 15 mmol) was dissolved in dry tetrahydrofuran (40 mL) at −78 °C under an argon atmosphere. Sodium hexamethyldisilazide (NaHMDS, 11.25 mL, 2.0 M in THF, 22.5 mmol) was added slowly and the resulting mixture was stirred for 1 h, MeI (4.67 mL, 75 mmol) was then added dropwise. The reaction solution was stirred for 2 h at −78 °C and warmed up to −60 °C to react for 3 h. The reaction was quenched with saturated aqueous NH_4_Cl solution (30 mL). The layers were separated, and the aqueous phase was extracted with ethyl acetate (3 × 50 mL). The combined organic layers were washed with brine (50 mL) and dried over anhydrous Na_2_SO_4_, concentrated under reduced pressure. The residue was purified by silica gel column chromatography (ethyl acetate/petroleum ether = 1:8) to provide (*R*)-4-isopropyl-3-((*R*)-2-methylhex-5-enoyl)oxazolidin-2-one (**7**) as a colorless oil (2.73 g, 76% yield). [α]_D_^25^ = −91.08 (c 0.55, CHCl_3_). ^1^H NMR (400 MHz, Chloroform-*d*) δ 5.83– 5.73 (m, 1H), 5.03–4.89 (m, 2H), 4.46–4.42 (m, 1H), 4.28–4.18 (m, 2H), 3.80–3.71 (m, 1H), 2.38–2.31 (m, 1H), 2.06 (q, *J* = 7.1 Hz, 2H), 1.90–1.81 (m, 1H), 1.52–1.45 (m, 1H), 1.21 (d, *J* = 6.9 Hz, 3H), 0.91 (d, *J* = 7.0 Hz, 3H), 0.87 (d, *J* = 6.9 Hz, 3H). ^13^C NMR (101 MHz, CDCl_3_) δ 177.17, 153.78, 138.30, 114.99, 63.37, 58.57, 37.34, 32.30, 31.68, 28.60, 18.08, 18.06, 14.85. HRMS (ESI, *m*/*z*): calculated for [M + Na]^+^ C_13_H_21_NO_3_Na 262.1414, found: 262.1417.

*Synthesis of (R)-2-methylhex-5-en-1-ol* (**8**)

LiAlH_4_ (1.50 g, 38.75 mmol) was dissolved in dry tetrahydrofuran (15 mL) at 0 °C under an argon atmosphere, the solution of (*R*)-4-isopropyl-3-((*R*)-2-methylhex-5-enoyl)oxazolidin-2-one (**7**) (2.65 g, 11.07 mmol) in dry tetrahydrofuran (10 mL) was then added dropwise over 20 min at 0 °C. The reaction mixture was warmed to 25 °C and stirred for 12 h, followed by the slow addition of saturated aqueous ammonium chloride (20 mL) and diluted with ethyl acetate. The precipitate was filtered off, and the filtrate was then dried over anhydrous sodium sulfate and filtered. The filtrate was concentrated under reduced pressure. The residue was purified by silica gel column chromatography (ethyl acetate/petroleum ether 1:5) to provide (*R*)-2-methylhex-5-en-1-ol (**8**) as a colorless oil (1.09 g, 86% yield, ≥99% ee, determined by ^1^H NMR analysis of the ester derived from (*S*)-MTPACl). [α]_D_^25^ = +7.17 (c 0.61, CHCl_3_). ^1^H NMR (400 MHz, Chloroform-*d*) δ 5.86–5.76 (m, 1H), 4.98 (dd, *J* = 27.7, 13.6 Hz, 2H), 3.53–3.41 (m, 2H), 2.18–2.00 (m, 2H), 1.70–1.59 (m, 1H), 1.56–1.47 (m, 1H), 1.41 (s, 1H), 1.25–1.16 (m, 1H), 0.93 (d, *J* = 6.7 Hz, 3H). ^13^C NMR (101 MHz, CDCl_3_) δ 139.03, 114.54, 68.31, 35.35, 32.43, 31.31, 16.57. HRMS (ESI, *m*/*z*): calculated for [M + K]^+^ C_7_H_14_OK 153.0676, found: 153.0662.

*Synthesis of (S)-5-methylundec-1-ene* (**10**)

Tosyl chloride (1.90 g, 10.0 mmol) in dry dichloromethane (20 mL) was added slowly to a solution of (*R*)-2-methylhex-5-en-1-ol (**8**) (0.57 g, 5.0 mmol) and triethylamine (1.24 mL, 8.75 mmol) in dichloromethane (10 mL) under an argon atmosphere. The mixture was stirred for 18 h at room temperature. Afterward, the reaction mixture was quenched with saturated aqueous sodium bicarbonate (20 mL), extracted with dichloromethane (3 × 30 mL), the combined organic layers were washed with brine (30 mL) and dried over anhydrous Na_2_SO_4_, concentrated under reduced pressure. The residue was purified by column chromatography on silica gel with ethyl acetate/petroleum ether (1:5) to yield the desired tosylate product as a colorless oil.

0.1 M solution of Li_2_CuCl_4_ in THF (5.0 mL, 0.5 mmol) and 1.0 M solution of pentylmagnesium bromide (**9**) (15.0 mL, 15.0 mmol) were added to a solution of the previously obtained tosylate (5.0 mmol, 1.0 equiv.) in dry tetrahydrofuran (10 mL) at –20 °C under an argon atmosphere. The mixture was allowed to warm up to room temperature and stirred overnight. The reaction was quenched with saturated aqueous NH_4_Cl solution (30 mL). The layers were separated, and the aqueous phase was extracted with ethyl acetate (3 × 40 mL). The combined organic layers were washed with brine (40 mL) and dried over anhydrous Na_2_SO_4_. The residue was purified by silica gel column chromatography (petroleum ether) to provide (*S*)-5-methylundec-1-ene (**10**) (0.70 g, 83% yield) as a colorless oil. [α]_D_^25^ = −1.22 (c 0.65, CHCl_3_). ^1^H NMR (400 MHz, Chloroform-*d*) δ 5.87–5.77 (m, 1H), 5.05–4.90 (m, 2H), 2.13–1.98 (m, 2H), 1.44–1.36 (m, 2H), 1.31–1.24 (m, 9H), 1.21–1.07 (m, 2H), 0.90–0.85 (m, 6H). ^13^C NMR (101 MHz, CDCl_3_) δ 139.65, 114.06, 37.11, 36.40, 32.44, 32.10, 31.55, 29.83, 27.13, 22.85, 19.68, 14.28. HRMS (ESI, *m*/*z*): calculated for [M + Na]^+^ C_12_H_24_Na 191.1770, found: 191.1786.

*Synthesis of (S)-5-methylundecan-1-ol* (**11**)

9-Borabicyclo [3.3.1] nonane (9-BBN) (22.8 mL, 0.5 M in THF, 11.4 mmol) was added to a solution of (*S*)-5-methylundec-1-ene (**10**) (0.64 g, 3.8 mmol) in dry tetrahydrofuran (10 mL) slowly at room temperature under an argon atmosphere. The reaction was stirred for 12 h, followed by the addition of sodium hydroxide solution (7.6 mL, 3 M, 22.8 mmo) at 0 °C. After stirring for 30 min, the temperature was lowered to –20 °C, and hydrogen peroxide solution (7.6 mL, 30%) was added slowly. The reaction was stirred for 3 h at room temperature and was quenched with saturated aqueous ammonium chloride (15 mL). The layers were separated, and the aqueous phase was extracted with ethyl acetate (3 × 50 mL). The combined organic layers were washed with brine (50 mL) and dried over anhydrous Na_2_SO_4_. The residue was purified by silica gel column chromatography (ethyl acetate/petroleum ether = 1:10) to provide (*S*)-5-methylundecan-1-ol (**11**) (0.57 g, 80% yield) as a colorless oil. [α]_D_^25^ = −1.41 (c 0.85, CHCl_3_). ^1^H NMR (400 MHz, Chloroform-d) δ 3.64 (t, *J* = 6.6 Hz, 2H), 1.62–1.48 (m, 2H), 1.43–1.19 (m, 14H), 1.16–1.04 (m, 2H), 0.94–0.79 (m, 6H). ^13^C NMR (101 MHz, CDCl_3_) δ 63.25, 37.16, 36.98, 33.30, 32.89, 32.09, 29.82, 27.17, 23.36, 22.84, 19.77, 14.26. HRMS (ESI, *m*/*z*): calculated for [M + Na]^+^ C_12_H_26_ONa 209.1876, found: 209.1882.

*Synthesis of (S)-1-iodo-5-methylundecane* (**12**)

To a stirred mixture of (*S*)-5-methylundecan-1-ol (**11**) (0.37 g, 2.0 mmol), triphenylphosphine (0.63 g, 2.4 mmol) and imidazole (0.30g, 4.4 mmol) in dry tetrahydrofuran (5 mL) were added to iodine (0.61 g, 2.4 mmol) in dry tetrahydrofuran (5 mL) at room temperature under an argon atmosphere. After 3 h, the reaction was quenched with 5% Na_2_S_2_O_3_ aqueous solution, and the resulting mixture was extracted with ethyl acetate (3 × 30 mL). The combined organic layers were washed with 5% Na_2_S_2_O_3_ aqueous solution, water and brine, dried over anhydrous Na_2_SO_4_. The residue was purified by silica gel column chromatography (petroleum ether) to provide (*S*)-1-iodo-5-methylundecane (**12**) (0.52 g, 88% yield) as a light-yellow oil. [α]_D_^25^ = +0.88 (c 0.90, CHCl_3_). ^1^H NMR (500 MHz, Chloroform-*d*) δ 3.19 (t, *J* = 7.0 Hz, 2H), 1.85–1.76 (m, 2H), 1.45–1.34 (m, 3H), 1.32–1.24 (m, 10H), 1.15–1.07 (m, 2H), 0.88 (t, *J* = 6.9 Hz, 3H), 0.85 (d, *J* = 6.6 Hz, 3H). ^13^C NMR (126 MHz, CDCl_3_) δ 37.11, 36.01, 34.02, 32.74, 32.09, 29.81, 28.16, 27.15, 22.84, 19.77, 14.27, 7.52. HRMS (ESI, *m*/*z*): calculated for [M + H]^+^ C_12_H_26_I 297.1074, found: 297.1063.

*Synthesis of (R)-6-iodo-5-methylhex-1-ene* (**13**)

Following the similar procedure for the synthesis of (*S*)-1-iodo-5-methylundecane (**12**). To a stirred mixture of (*R*)-2-methylhex-5-en-1-ol (**8**) (0.29 g, 2.5 mmol), triphenylphosphine (0.79 g, 3.0 mmol) and imidazole (0.37g, 5.5 mmol) in dry tetrahydrofuran (8 mL) was added iodine (0.76 g, 3.0 mmol) in dry tetrahydrofuran (8 mL) at room temperature under an argon atmosphere. After 3 h, the reaction was quenched with 5% Na_2_S_2_O_3_ aqueous solution, and the resulting mixture was extracted with ethyl acetate (3 × 40 mL). The combined organic layers were washed with 5% Na_2_S_2_O_3_ aqueous solution, water and brine, dried over anhydrous Na_2_SO_4_. The residue was purified by silica gel column chromatography (petroleum ether) to provide (*R*)-6-iodo-5-methylhex-1-ene (**13**) (0.45 g, 80% yield) as a light-yellow oil. [α]_D_^25^ = −2.90 (c 0.83, CHCl_3_). ^1^H NMR (500 MHz, Chloroform-*d*) δ 5.83–5.75 (m, 1H), 5.05–5.01 (m, 1H), 4.99–4.95 (m, 1H), 3.24 (dd, *J* = 9.6, 4.4 Hz, 1H), 3.17 (dd, *J* = 9.7, 5.7 Hz, 1H), 2.09–2.04 (m, 2H), 1.52–1.45 (m, 2H), 1.35–1.27 (m, 1H), 0.99 (d, *J* = 6.4 Hz, 3H). ^13^C NMR (126 MHz, CDCl_3_) δ 138.40, 114.97, 35.70, 34.17, 31.24, 20.60, 17.75. HRMS (ESI, *m*/*z*): calculated for [M + H]^+^ C_17_H_14_I 225.0135, found: 225.0145.

*Synthesis of (R)-1-methyl-4-((2-methylhex-5-en-1-yl)sulfonyl)benzene* (**14**)

To a stirred mixture of sodium *p*-toluenesulfinate (0.25 g, 1.4 mmol), PEG-400 (3 mL), and DMSO (1.5 mL) was added (*R*)-6-iodo-5-methylhex-1-ene (**13**) (0.22 g, 1.0 mmol) at room temperature under an argon atmosphere. The mixture was warmed to 80 °C and stirred for 2 h. The reaction was quenched with a 10% NaCl solution (10 mL) after cooling to room temperature. The crude product was extracted with ethyl acetate (3 × 40 mL). The combined organic layers were washed with water and brine, dried over Na_2_SO_4_, and concentrated in vacuo. The residue was purified by silica gel column chromatography (ethyl acetate/petroleum ether = 1:8) to provide (*R*)-1-methyl-4-((2-methylhex-5-en-1-yl)sulfonyl)benzene (**14**) (0.18 g, 70% yield) as a colorless oil. [α]_D_^25^ = −3.06 (c 0.95, CHCl_3_). ^1^H NMR (500 MHz, Chloroform-*d*) δ 7.78 (d, *J* = 8.2 Hz, 2H), 7.35 (d, *J* = 8.0 Hz, 2H), 5.75–5.66 (m, 1H), 4.99–4.89 (m, 2H), 3.07 (dd, *J* = 14.2, 4.7 Hz, 1H), 2.91 (dd, *J* = 14.2, 7.7 Hz, 1H), 2.45 (s, 3H), 2.15–2.05 (m, 1H), 2.04–1.92 (m, 2H), 1.56–1.49 (m, 1H), 1.37–1.29 (m, 1H), 1.07 (d, *J* = 6.7 Hz, 3H). ^13^C NMR (126 MHz, CDCl_3_) δ 144.63, 137.95, 137.36, 130.02, 128.04, 115.15, 62.75, 35.93, 30.76, 28.29, 21.76, 19.87. HRMS (ESI, *m*/*z*): calculated for [M + H]^+^ C_14_H_21_O_2_S 253.1257, found: 253.1259.

*Synthesis of 1-(((5R,11S)-5,11-dimethylheptadec-1-en-6-yl)sulfonyl)-4-methylbenzene* (**15**)

To a stirred solution of (*R*)-1-methyl-4-((2-methylhex-5-en-1-yl)sulfonyl)benzene (**14**) (0.15 g, 0.6 mmol) in dry tetrahydrofuran (10 mL) was added *n*-BuLi (2.4 M, 0.38 mL, 0.9 mmol) slowly at –78 °C under an argon atmosphere. The reaction mixture was stirred at –78 °C for 30 min and then at –35 °C for 30 min. After cooling to –78 °C, (*S*)-1-iodo-5-methylundecane (**12**) (0.21 g, 0.72 mmol) dissolved in HMPA (0.42 mL) and dry tetrahydrofuran (1 mL) was added dropwise. The reaction was then allowed to warm gradually to room temperature and stirred for overnight. The resulting mixture was poured into 1 M HCl (6 mL) and extracted with ethyl acetate (3 × 20 mL). The combined organic layers were washed with a saturated NaHCO_3_ solution and brine, dried over Na_2_SO_4_, and concentrated in vacuo. The residue was purified by silica gel column chromatography (ethyl acetate/petroleum ether = 1:10) to provide 1-(((5*R*,11*S*)-5,11-dimethylheptadec-1-en-6-yl)sulfonyl)-4-methylbenzene (**15**) (0.19 g, 75% yield) as a colorless oil. [α]_D_^25^ = −0.38 (c 1.04, CHCl_3_). ^1^H NMR (500 MHz, Chloroform-*d*) δ 7.75 (d, *J* = 8.1 Hz, 2H), 7.34 (d, *J* = 8.0 Hz, 2H), 5.80–5.62 (m, 1H), 5.01–4.89 (m, 2H), 2.91–2.85 (m, 1H), 2.45 (s, 3H), 2.23–2.10 (m, 1H), 2.04–1.81 (m, 3H), 1.66–1.61 (m, 1H), 1.40–1.35 (m, 1H), 1.33–1.11 (m, 17H), 1.04–1.00 (m, 3H), 0.88 (t, *J* = 6.9 Hz, 3H), 0.79 (d, *J* = 6.5 Hz, 3H). ^13^C NMR (126 MHz, CDCl_3_) δ 144.39, 137.91 (138.50), 136.54 (136.86), 129.86 (129.83), 128.73 (128.70), 115.18 (114.97), 68.30 (69.70), 37.16 (37.13), 36.69 (36.72), 35.18, 32.73 (32.78), 32.27, 32.09 (31.99), 31.59, 31.48 (31.06), 29.81, 29.49 (29.19), 27.16 (27.04), 25.14, 22.84 (23.92), 21.74, 19.74 (18.09), 14.85, 14.27. HRMS (ESI, *m*/*z*): calculated for [M + H]^+^ C_26_H_45_O_2_S 421.3135, found: 421.3117.

*Synthesis of (5R,11S)-5,11-dimethylheptadecane* (**1**)

To a stirred solution of magnesium powder (0.13 g, 5.4 mmol) in dry tetrahydrofuran (1.0 mL), a catalytic amount of MeMgBr (3.0 M, two drops) was added to activate the metal under an argon atmosphere. After stirring for 15 min at room temperature, a solution of 1-(((5*R*,11*S*)-5,11-dimethylheptadec-1-en-6-yl)sulfonyl)-4-methylbenzene (**15**) (0.15 g, 0.35 mmol) in dry MeOH (5.0 mL) was added, the mixture was then stirred at 50 °C for 4 h. Next, the mixture was evaporated to remove MeOH and an ice-cooled 1 M HCl solution (2 mL) was added to dissolve the residues. The crude products were extracted with Et_2_O (3 × 10 mL), washed with saturated NaHCO_3_ solution and brine, dried over Na_2_SO_4_, and concentrated in vacuo. The residue was filtered through a silica gel pad and concentrated in vacuo to afford the crude product. The crude product was dissolved in 3 mL MeOH and added to a suspension of palladium/carbon (30 mg, 10% wt.) in MeOH (2 mL) at room temperature under a hydrogen atmosphere. The reaction mixture was stirred for 12 h and concentrated in vacuo. The residue was purified by silica gel column chromatography (petroleum ether) to provide (5*R*,11*S*)-5,11-dimethylheptadecane (**1**) (63.9 mg, 68% yield) as a colorless oil. [α]_D_^25^ = −0.93 (c 0.63, CHCl_3_). ^1^H NMR (500 MHz, Chloroform-*d*) δ 1.41–1.33 (m, 2H), 1.31–1.21 (m, 22H), 1.14–1.03 (m, 4H), 0.91–0.87 (m, 6H), 0.86–0.81 (m, 6H). ^13^C NMR (126 MHz, CDCl_3_) δ 37.27, 36.94, 32.92, 32.90, 32.13, 30.54, 30.20, 29.90, 29.86, 29.51, 27.28, 27.21, 23.21, 22.86, 19.89, 14.33, 14.28. HRMS (ESI, *m*/*z*): calculated for [M + K]^+^ C_19_H_40_K 307.2762, found:307.2751.

*Synthesis of (S)-7-methylheptadecane* (**2**)

Following the similar procedure for the synthesis of (*S*)-5-methylundec-1-ene (**10**). Tosyl chloride (0.19 g, 1.0 mmol) in dry dichloromethane (2 mL) was added slowly to a solution of (*S*)-5-methylundecan-1-ol (**11**) (0.57 g, 0.5 mmol) and triethylamine (0.12 mL, 0.88 mmol) in dichloromethane (2 mL) under an argon atmosphere. The mixture was stirred for 18 h at room temperature. Afterward, the reaction mixture was quenched with saturated aqueous sodium bicarbonate (4 mL), extracted with dichloromethane (3 × 15 mL), the combined organic layers were washed with brine (20 L) and dried over anhydrous Na_2_SO_4_, concentrated under reduced pressure. The residue was purified by column chromatography on silica gel with ethyl acetate/petroleum ether (1:5) to yield the desired tosylate product as a colorless oil.

0.1 M solution of Li_2_CuCl_4_ in THF (0.5 mL, 0.05 mmol) and 1.0 M solution of hexylmagnesium bromide (**16**) (1.5 mL, 1.5 mmol) were added to a solution of the previously obtained tosylate (0.5 mol, 1.0 equiv.) in dry tetrahydrofuran (2 mL) at –20 °C under an argon atmosphere. The mixture was allowed to warm up to room temperature and stirred overnight. The reaction was quenched with saturated aqueous NH_4_Cl solution (5 mL). The layers were separated, and the aqueous phase was extracted with ethyl acetate (3 × 30 mL). The combined organic layers were washed with brine (30 mL) and dried over anhydrous Na_2_SO_4_. The residue was purified by silica gel column chromatography (petroleum ether) to provide (*S*)-7-methylheptadecane (**2**) (98 mg, 77% yield) as a colorless oil. [α]_D_^25^ = −1.76 (c 0.87, CHCl_3_). ^1^H NMR (400 MHz, Chloroform-*d*) δ 1.38–1.03 (m, 28H), 1.09–1.04 (m, 1H), 0.90–0.82 (m, 9H). ^13^C NMR (101 MHz, CDCl_3_) δ 37.27, 32.92, 32.13, 32.10, 30.21, 29.90, 29.87, 29.83, 29.53, 27.25, 27.22, 22.86, 19.88, 14.28. HRMS (ESI, *m*/*z*): calculated for [M + K]^+^ C_18_H_38_K 293.2605, found: 293.2601.

*Synthesis of (S)-4-isopropyl-3-tetradecanoyloxazolidin-2-one* (**19**)

Following the similar procedure for the synthesis of (*R*)-3-(hex-5-enoyl)-4-isopropyloxazolidin-2-one (**6**), tetradecanoic acid (**17**) (0.91 g, 4 mmol) and (*S*)-4-isopropyloxazolidin-2-one (0.67 g, 5.2 mmol) were reacted to afford (*S*)-4-isopropyl-3-tetradecanoyloxazolidin-2-one (**19**) (1.15 g, 85% yield) as a white solid. The melting point was 56–57 °C; [α]_D_^25^ = +56.03 (c 3.02, CHCl_3_). ^1^H NMR (500 MHz, Chloroform-*d*) δ 4.44–4.41 (m, 1H), 4.25 (t, *J* = 8.7 Hz, 1H), 4.19 (dd, *J* = 9.1, 3.0 Hz, 1H), 3.00–2.94 (m, 1H), 2.87–2.81 (m, 1H), 2.41–2.32 (m, 1H), 1.69–1.59 (m, 2H), 1.35–1.24 (m, 20H), 0.90 (d, *J* = 7.1 Hz, 3H), 0.88–0.86 (m, 6H). ^13^C NMR (126 MHz, CDCl_3_) δ 173.56, 154.20, 63.42, 58.50, 35.65, 32.04, 29.80, 29.77, 29.73, 29.61, 29.50, 29.48, 29.26, 28.51, 24.60, 22.81, 18.10, 14.78, 14.24. HRMS (ESI, *m*/*z*): calculated for [M + K]^+^ C_20_H_37_O_3_NK 378.2405, found: 378.2405.

*Synthesis of (S)-4-isopropyl-3-((S)-2-methyltetradecanoyl)oxazolidin-2-one* (**20**)

Following the similar procedure for the synthesis of (*R*)-4-isopropyl-3-((*R*)-2-methylhex-5-enoyl)oxazolidin-2-one (**7**), (*S*)-4-isopropyl-3-tetradecanoyloxazolidin-2-one (**19**) (1.02 g, 3 mmol) and methyl iodide (0.93 mL, 15 mmol) were reacted to afford (*S*)-4-isopropyl-3-((*S*)-2-methyltetradecanoyl)oxazolidin-2-one (**20**) (0.83 g, 78% yield) as a colorless oil. [α]_D_^25^ = +60.61 (c 1.96 CHCl_3_). ^1^H NMR (500 MHz, Chloroform-*d*) δ 4.46–4.43 (m, 1H), 4.25 (t, *J* = 8.7 Hz, 1H), 4.20 (dd, *J* = 9.1, 2.9 Hz, 1H), 3.75–3.68 (m, 1H), 2.38–2.32 (m, 1H), 1.74–1.66 (m, 1H), 1.38–1.32 (m, 1H), 1.30– 1.23 (m, 20H), 1.19 (d, *J* = 6.9 Hz, 3H), 0.91 (d, *J* = 7.0 Hz, 3H), 0.89–0.86 (m, 6H). ^13^C NMR (126 MHz, CDCl_3_) δ 177.46, 153.80, 63.32, 58.57, 37.86, 33.26, 32.06, 29.81, 29.81, 29.78, 29.73, 29.65, 29.50, 28.57, 27.45, 22.82, 18.08, 17.99, 14.82, 14.25. HRMS (ESI, *m*/*z*): calculated for [M + Na]^+^ C_21_H_39_O_3_NNa 378.2822, found: 378.2813.

*Synthesis of (S)-2-methyltetradecan-1-ol* (**21**)

Following the similar procedure for the synthesis of (*R*)-2-methylhex-5-en-1-ol (**8**), (*S*)-4-isopropyl-3-((*S*)-2-methyltetradecanoyl)oxazolidin-2-one (**20**) (0.64 g, 1.8 mmol) and LiAlH_4_ (0.24 g, 6.3 mmol) were reacted to afford (*S*)-2-methyltetradecan-1-ol (**21**) (0.35 g, 81% yield, ≥99% ee, determined by ^1^H NMR analysis of the ester derived from (*S*)-MTPACl) as a colorless oil. [α]_D_^25^= −10.60 (c 1.10, CHCl_3_). ^1^H NMR (500 MHz, Chloroform-*d*) δ 3.51 (dd, *J* = 10.5, 5.8 Hz, 1H), 3.42 (dd, *J* = 10.5, 6.6 Hz, 1H), 1.64–1.57 (m, 1H), 1.39–1.35 (m, 2H), 1.31–1.26 (s, 20H), 1.13–1.07 (m, 1H), 0.91 (d, *J* = 6.7 Hz, 3H), 0.88 (t, *J* = 7.0 Hz, 3H). ^13^C NMR (126 MHz, CDCl_3_) δ 68.58, 35.92, 33.30, 32.07, 30.10, 29.83, 29.81, 29.80, 29.51, 27.13, 22.84, 16.73, 14.26. HRMS (ESI, *m*/*z*): calculated for [M + Na]^+^ C_15_H_32_ONa 251.2345, found: 251.2364.

*Synthesis of (S)-2,5-dimethylheptadecane* (**3**)

Following the similar procedure for the synthesis of (*S*)-5-methylundec-1-ene (**10**), (*S*)-2-methyltetradecan-1-ol (**21**) (0.18 g, 0.8 mmol) and isobutylmagnesium bromide (**22**) (4.8 mL, 0.5 M in THF, 2.4 mmol) were reacted to afford (*S*)-2,5-dimethylheptadecane (**3**) (0.15 g, 72% yield) as a colorless oil. [α]_D_^25^ = −0.77 (c 1.04, CHCl_3_). literature value: [α]_D_^18^ = −0.37 (c 2.98, Hexane). ^1^H NMR (400 MHz, Chloroform-*d*) δ 1.51–1.43 (m, 1H), 1.34–1.23 (m, 23H), 1.18–1.05 (m, 4H), 0.90–0.83 (m, 12H). ^13^C NMR (101 MHz, CDCl_3_) δ 37.27, 36.55, 34.93, 33.18, 32.10, 30.21, 29.90, 29.87, 29.83, 29.53, 28.50, 27.26, 22.99, 22.86, 22.76, 19.92, 14.28. HRMS (ESI, *m*/*z*): calculated for [M + K]^+^ C_19_H_32_NO_3_ 307.2762, found: 307.2758.

### 4.2. Behavioral Assays: Y-Tube Olfactometer and Cage Tests

#### 4.2.1. Insects

Pupae of male *S. cinerearia* were collected from Sishui, Shandong Province, China, and reared to adult emergence at the Institute of Industrial Crops, Shandong Academy of Agricultural Sciences. Insects were maintained under controlled environmental conditions at a temperature of 25 ± 1 °C, with a relative humidity of 70–80%, and a photoperiod of 16 h light/8 h dark. Only healthy, active and unmated males, 2 days post-eclosion with an average body length of approximately 12–14 mm and a body weight of 15–20 mg were selected for behavioral assays.

#### 4.2.2. Preparation of Pheromone Component Solutions

Each pheromone component (compounds **1**–**3**) was synthesized as described above and stored at –20 °C until use. Stock solutions were prepared in high-purity hexane (HPLC grade) at a concentration of 1 mg/mL. Working solutions of the desired doses (1 µg, 10 µg, 100 µg, and 1000 µg) were obtained by serial dilution with the same solvent. For behavioral assays, aliquots (10 µL) of each working solution were pipetted onto a 1 × 0.5 cm piece of filter paper, which was allowed to air dry for 2 min before being placed in the Y-tube olfactometer or attached inside the cage bioassay setup. Filter papers treated with hexane alone served as solvent control.

#### 4.2.3. Y-Tube Olfactometer Assay

Behavioral choices were measured in a glass Y-tube (common arm: 15 cm; side arms: 17 cm; I.D. 2.5 cm; 60° bifurcation). Charcoal-filtered, humidified air was pulled through each arm at 200 mL min^−1^ (verified with a flowmeter). One arm received the treatment lure; the other received a solvent control. A single male *S. cinerearia* was released at the base of the common arm; a choice was scored when the moth moved ≥5 cm into an arm and remained for ≥30 s within a 3 min limit. Non-responders were recorded and excluded from choice analyses but reported as no choice. Between replicates, glassware was rinsed with hexane and baked (120 °C, ≥1 h). For each stimulus (or dose), 50 males were tested (3 biological replicates).

#### 4.2.4. Cage Bioassay

Semi-field attraction was evaluated in mesh cages (60 × 60 × 50 cm). A treatment lure (Compound **1** at 1, 10, 100, or 1000 μg) and a solvent control were put at opposite corners, 50–60 cm apart. Groups of 20 males *S. cinerearia* were released from the cage center. After 10, 20, 30 min, the number of males within a 10 cm radius of each lure was recorded; the final read at 30 min was used for analysis (3 biological replicates).

#### 4.2.5. Data Analysis

For the Y-tube and cage bioassays, treatment versus control choices were analyzed using SPSS software (version 23.0; IBM Corporation, Chicago, IL, USA). Comparisons between treatment and control groups were performed using Student’s *t*-test, with statistical significance defined as *p* < 0.05 (*) and *p* < 0.01 (**).

## 5. Conclusions

In summary, our work demonstrates both a methodological advance in asymmetric synthesis and a novel ecological finding in insect chemical communication. A concise and efficient stereoselective synthetic strategy was established for three sex pheromone components of *L. fiscellaria lugubrosa*, achieving excellent enantiomeric purity and improved overall yields. Importantly, behavioral assays revealed that compound **1** exhibits attractive effects on male *S. cinerearia*, with dose-dependent responses validated in both Y-tube and cage experiments. These results not only expand our understanding of pheromone-mediated communication within the Geometridae but also suggest new avenues for pest management. Collectively, this study bridges advances in synthetic chemistry with insect chemical ecology, highlighting the potential of stereoselectively synthesized pheromone components to uncover novel biological phenomena and support practical applications in integrated pest control.

## Data Availability

The data presented in this article are available in the Supplementary Materials.

## Supplementary Materials

The following supporting information can be downloaded at: https://www.mdpi.com/article/10.3390/molecules30214216/s1, the detailed synthetic procedure for compound **1**,**2** and **3**; ^1^H NMR and ^13^C NMR spectra for all the synthetic compounds and synthetic procedure of mosher esters of chiral primary alcohols **8** and **21**; Experimental data of compounds **1**, **2**, and **3** on male *S. cinerearia* in Y-tube and cage assays.
