# Correction: Zhou et al. Modular Stereoselective Synthesis of Sex Pheromone of *Lambdina fiscellaria lugubrosa* (Hulst) and Discovery of Cross-Species Attraction in *Semiothisa cinerearia* (Bremer & Grey). *Molecules* 2025, *30*, 4216

**DOI:** 10.3390/molecules30234492

**Published:** 2025-11-21

**Authors:** Yun Zhou, Jionglin Wang, Yueru Zhang, Xiaochen Fu, Xiaoyang Li, Jianan Wang, Xianchang Wang, Jianhua Zhang, Yanbing Gu, Jinlong Han, Jiangchun Zhong, Chenggang Shan

**Affiliations:** 1 Institute of Industrial Crops, Shandong Academy of Agricultural Sciences, Jinan 250100, China; zysass2021@163.com (Y.Z.); archangw@163.com (X.W.); zhangjianhua198904@163.com (J.Z.); goldendragonh@163.com (J.H.); 2 Department of Applied Chemistry, China Agricultural University, Beijing 100193, China; s20223102112@cau.edu.cn (J.W.); b20233100844@cau.edu.cn (X.L.); xxjnwang@163.com (J.W.); 3 School of Pharmacy, Shandong University of Traditional Chinese Medicine, Jinan 250355, China; 13553169667@163.com (Y.Z.); fxc13345203071@163.com (X.F.); 4 Sishui County Agricultural Technology Extension Center, Sishui 273200, China; guyanbing2010@163.com

## Error in Citation

In the original publication [1], References 1 and 2 were cited incorrectly. The correct Reference 1 is “Thomson, M.G. Appraisal of western hemlock looper infestations. *For. Chron.* **1957**, *33*, 141–147. https://doi.org/10.5558/tfc33141-2.”. The correct Reference 2 is “McCloskey, S.P.J.; Daniels, L.D.; McLean, J.A. Potential Impacts of Climate Change on Western Hemlock Looper Outbreaks. *Northwest Sci.* **2009**, *83*, 225–238. https://doi.org/10.3955/046.083.0306.”

The authors state that the scientific conclusions are unaffected. This correction was approved by the Academic Editor. The original publication has also been updated.
