# Supplementary material for: Modular Stereoselective Synthesis of Sex Pheromone of Lambdina fiscellaria lugubrosa (Hulst) and Discovery of Cross-Species Attraction in Semiothisa cinerearia (Bremer & Grey)
Source: Molecules. 2025 Oct 28;30(21):4216. doi: 10.3390/molecules30214216 (PMC12610577; doi:10.3390/molecules30214216)
Supplement: Supplementary file 1 [file molecules-30-04216-s001.zip › molecules-3923083-supplementary.pdf]

SUPPORTING INFORMATION

Modular Stereoselective Synthesis of Sex Pheromone of *Lambdina fiscellaria lugubrosa* (Hulst) and Discovery of Cross-Species Attraction in *Semiothisa cinerearia* (Bremer & Grey)

Yun Zhou [1], Jionglin Wang [2], Yueru Zhang [3], Xiaochen Fu [3], Xiaoyang Li [2], Jianan Wang [2], Xianchang Wang [1], Jianhua Zhang [1], Yanbing Gu [4], Jinlong Han [1], Jiangchun Zhong [2,*] and Chenggang Shan [1,*]

[1] Institute of Industrial Crops, Shandong Academy of Agricultural Sciences, Jinan 250100, China; zysass2021@163.com (Y.Z.); archangw@163.com (X.W.); zhangjianhua198904@163.com (J.Z.); goldendragonh@163.com (J.H.)

[2] Department of Applied Chemistry, China Agricultural University, Beijing 100193, China; s20223102112@cau.edu.cn (J.W.); b20233100844@cau.edu.cn (X.L.); xxjnwang@163.com (J.W.)

[3] School of Pharmacy, Shandong University of Traditional Chinese Medicine, Jinan 250355, China; 13553169667@163.com (Y.Z.); fxc13345203071@163.com (X.F.)

[4] Sishui County Agricultural Technology Extension Center, Sishui 273200, China; guyanbing2010@163.com

* Correspondence: zhong@cau.edu.cn (J.Z.); shanchenggang@126.com (C.S.); Tel.: +86-0531-6665-5005 (C.S.)

Table of Contents

1. General information S3

2. Synthesis of Mosher esters..S4

3. ^{1}H, ^{13}C spectra of the products..S20

4. Y-tube and cage experimental data...S22

5. Reference...S23

1. General information

All reactions were performed under an inert atmosphere of argon, utilizing a Schlenk line system. Reagents were sourced commercially and used without further purification, while solvents underwent distillation before application, following standard protocols. Proton NMR (^1H NMR) spectra were obtained at 500 MHz using TMS at δ 0.00 ppm or $CDCl_3$ at δ 7.26 ppm, and carbon NMR (^{13}C NMR) spectra were recorded at 126 MHz, with $CDCl_3$ set at δ 77.16 ppm as the internal standard, employing a Bruker DP-X500 spectrometer (Bruker Corporation, Beijing, China). High-resolution mass spectrometry (HRMS) data were collected using a Waters LCT Premier™ system (Waters Corporation, Beijing, China) equipped with an electrospray ionization (ESI) source. Optical rotation measurements were taken with a Rudolph Research Analytical AUTOPOL-IV polarimeter (Rudolph Research Analytical, Beijing, China). Melting points were determined using a Stuart SMP3 Melt-Temp apparatus (Stuart Equipment, Beijing, China) and are reported without correction.

2. Synthesis of Mosher esters

Scheme S1. Synthesis of Mosher esters **23-24**.

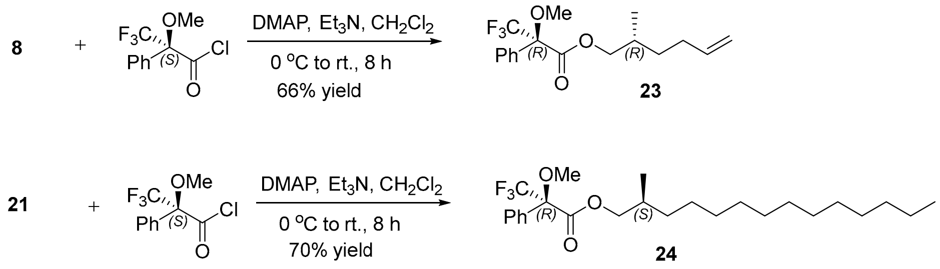

*(R)-2-methylhex-5-en-1-yl (R)-3,3,3-trifluoro-2-methoxy-2-phenylpropanoate (**23**)*

In an inert argon atmosphere, a solution containing DMAP (12.2 mg, 0.1 mmol, 1.0 equiv.) and triethylamine (50.6 mg, 0.5 mmol, 5.0 equiv.) in 4 mL of dichloromethane was prepared at 0 °C. To this solution, (*R*)-2-methylhex-5-en-1-ol (**8**) (11.4 mg, 0.1 mmol, 1.0 equiv.) was added gradually. Following this, (S)-(-)-α-methoxy-α-(trifluoromethyl)phenylacetyl chloride (50.6 mg, 0.2 mmol, 2.0 equiv.) was introduced, resulting in a yellow coloration of the reaction mixture. The reaction was then allowed to reach room temperature and stirred continuously overnight. Quenching of the reaction was achieved by the addition of 3 mL of water, and the organic phase was subsequently separated. The aqueous layer underwent extraction with dichloromethane (3 × 10 mL). The combined organic extracts were washed with brine (10 mL), dried over anhydrous sodium sulfate, and concentrated under reduced pressure to obtain the crude product. Finally, purification via thin-layer chromatography yielded (*R*)-2-methylhex-5-en-1-yl (*R*)-3,3,3-trifluoro-2-methoxy-2-phenylpropanoate (**23**) (21.8 mg, 66% yield) as a colorless oil [1]. ^{1}H NMR (400 MHz, Chloroform-*d*) δ 7.53 – 7.51 (m, 2H), 7.41 – 7.40 (m, 3H), 5.80 – 5.70 (m, 1H), 5.02 – 4.94 (m, 2H), 4.24 (dd, *J* = 10.7, 5.6 Hz, 1H), 4.10 (dd, *J* = 10.7, 6.5 Hz, 1H), 3.55 (s, 3H), 2.14 – 1.99 (m, 2H), 1.92 – 1.84 (m, 1H), 1.51 – 1.42 (m, 1H), 1.31 – 1.22 (m, 1H), 0.93 (d, *J* = 6.8 Hz, 3H). HRMS (ESI, *m*/*z*): calculated for [M + H]$^+$ $C_{17}H_{22}F_3O_3$ 331.1510, found: 331.1516.

*(S)-2-methyltetradecyl (R)-3,3,3-trifluoro-2-methoxy-2-phenylpropanoate (**24**)*

In accordance with the previously established method for synthesizing compound **23,** (*S*)-2-methyltetradecan-1-ol (**21**) (22.8 mg, 0.1 mmol, 1.0 equiv.) and (*S*)-(-)-α-methoxy-α-(trifluoromethyl)phenylacetyl chloride (50.6 mg, 0.2 mmol, 2.0 equiv.) were reacted to give (*S*)-2-methyltetradecyl (*R*)-3,3,3-trifluoro-2-methoxy-2-phenylpropanoate (**24**) (31.1 mg, 70% yield) as a colorless oil. ^{1}H NMR (500 MHz, Chloroform-d) δ 7.46 – 7.44 (m, 2H), 7.34 – 7.31 (m, 3H), 4.08 (dd, J = 6.1, 1.3 Hz, 2H), 3.48 (s, 3H), 1.80 – 1.74 (m, 1H), 1.24 – 1.19 (m, 22H), 1.08 – 1.19 (m, 1H), 0.84 (d, J = 6.7 Hz, 3H), 0.80 (d, J = 7.1 Hz, 3H). HRMS (ESI, *m*/*z*): calculated for [M + K]$^+$ $C_{25}H_{39}F_3O_3K$ 483.2483, found: 483.2477.

3. ^1H and ^{13}C spectra of the products

Figure S1. ^1H NMR Spectrum of compound **6** (400 MHz, $CDCl_3$)

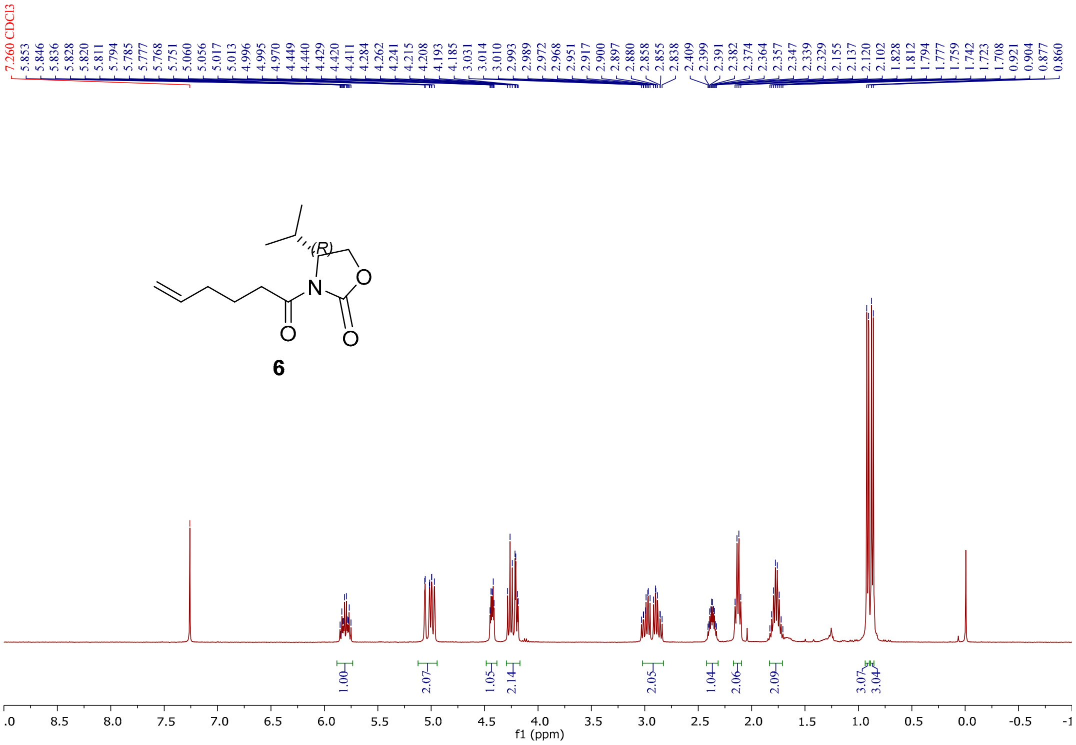

Figure S2. ^{13}C NMR Spectrum of compound **6** (101 MHz, $CDCl_3$)

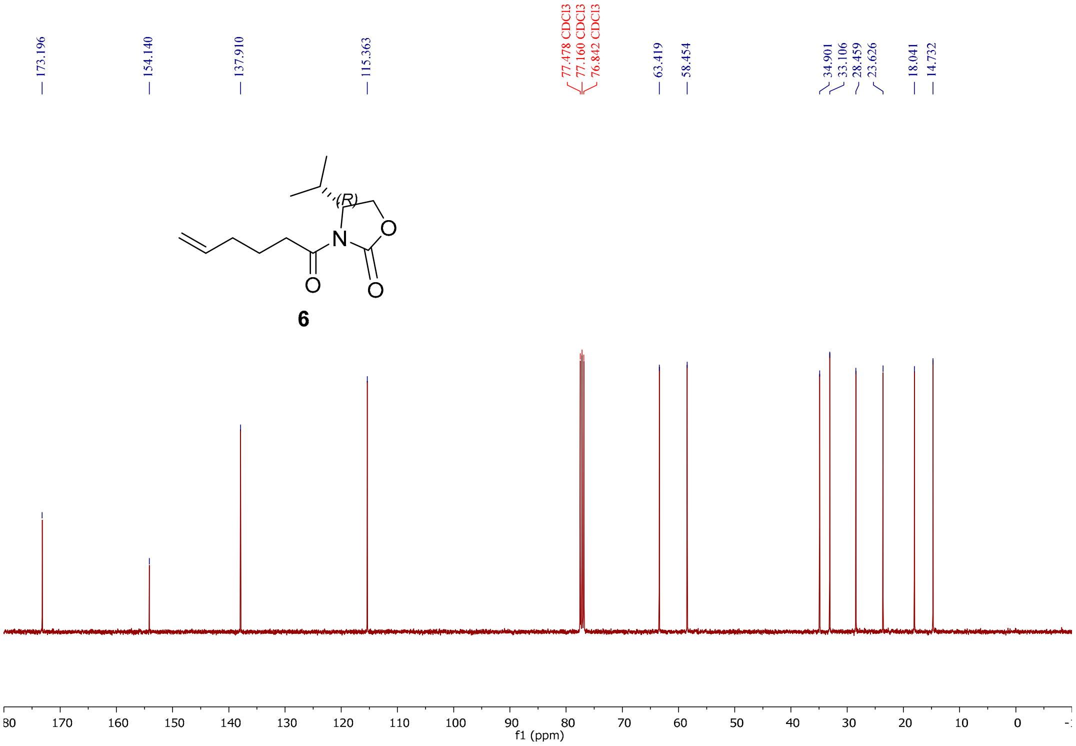

Figure S3. ^{1}H NMR Spectrum of compound **7** (400 MHz, $CDCl_3$)

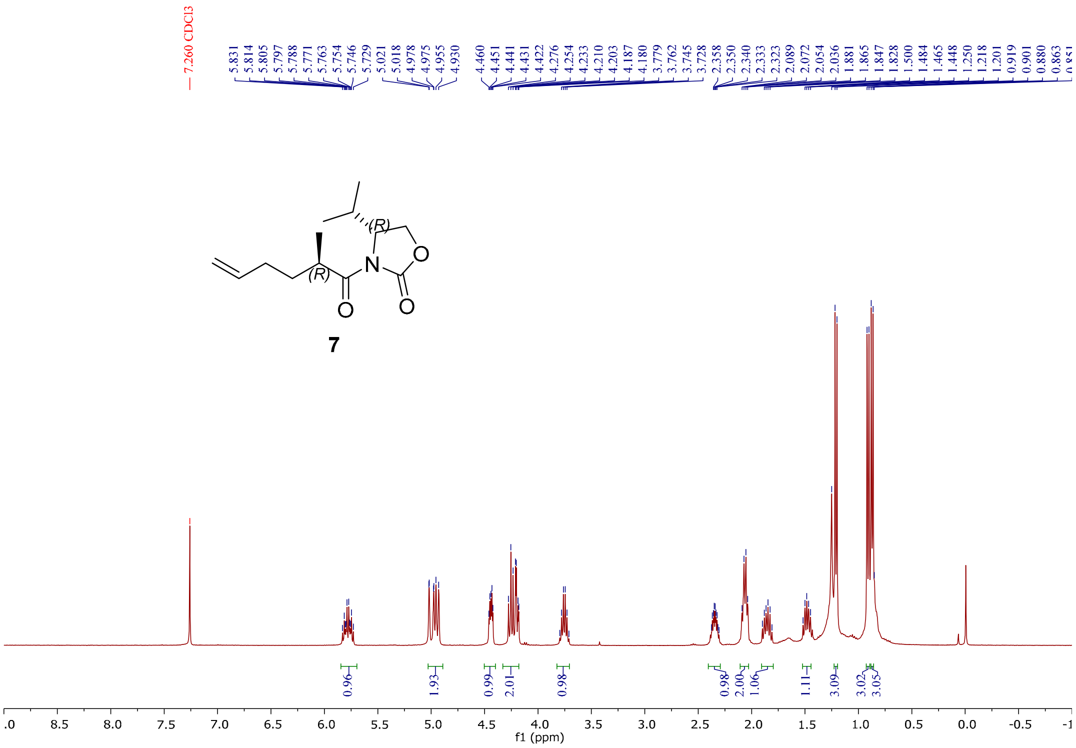

Figure S4. ^{13}C NMR Spectrum of compound **7** (101 MHz, $CDCl_3$)

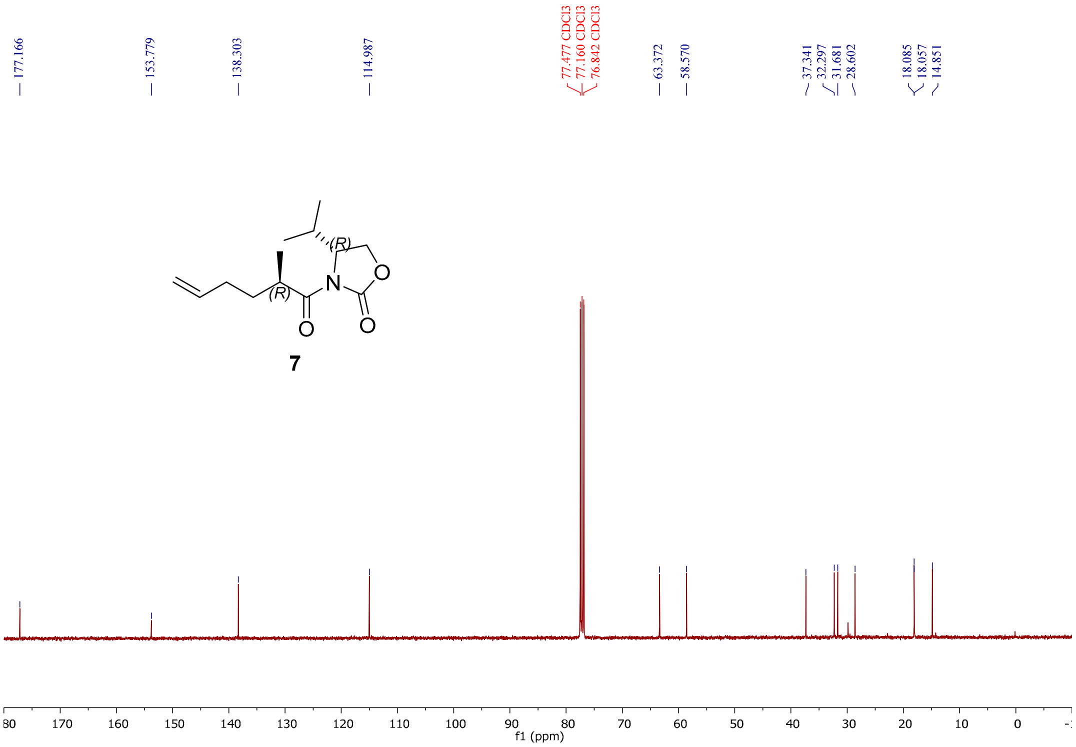

Figure S5. ^{1}H NMR Spectrum of compound **8** (400 MHz, $CDCl_3$)

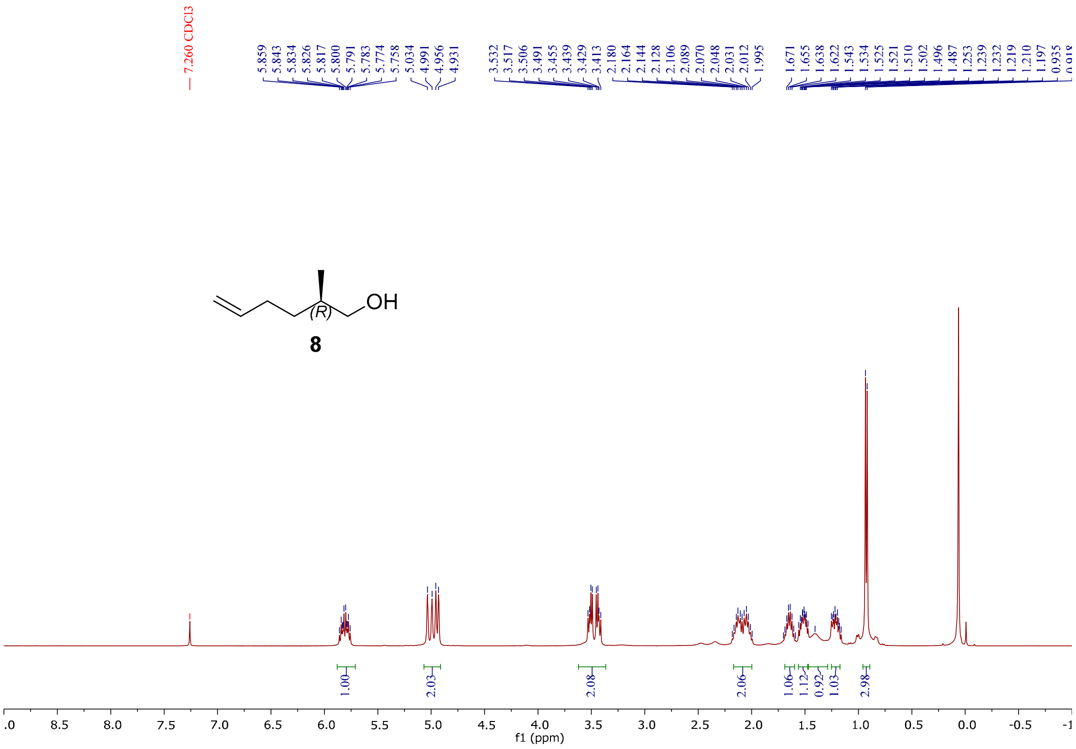

Figure S6. ^{13}C NMR Spectrum of compound **8** (101 MHz, $CDCl_3$)

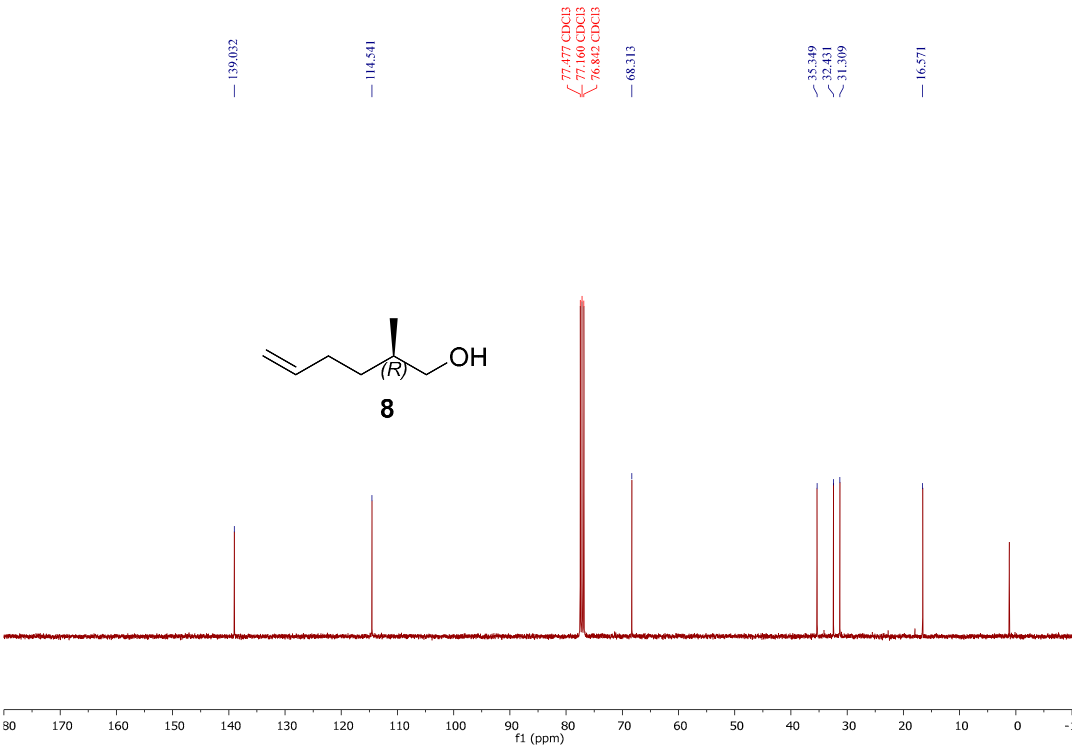

Figure S7. ^{1}H NMR Spectrum of compound **10** (400 MHz, $CDCl_3$)

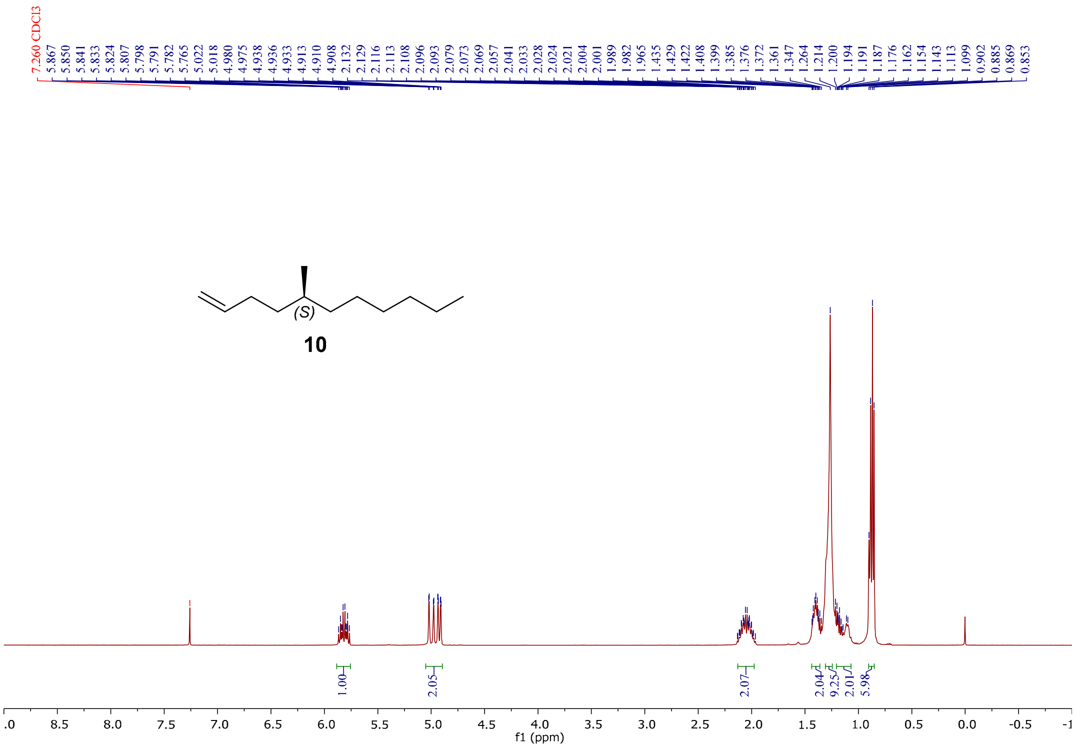

Figure S8. ^{13}C NMR Spectrum of compound **10** (101 MHz, $CDCl_3$)

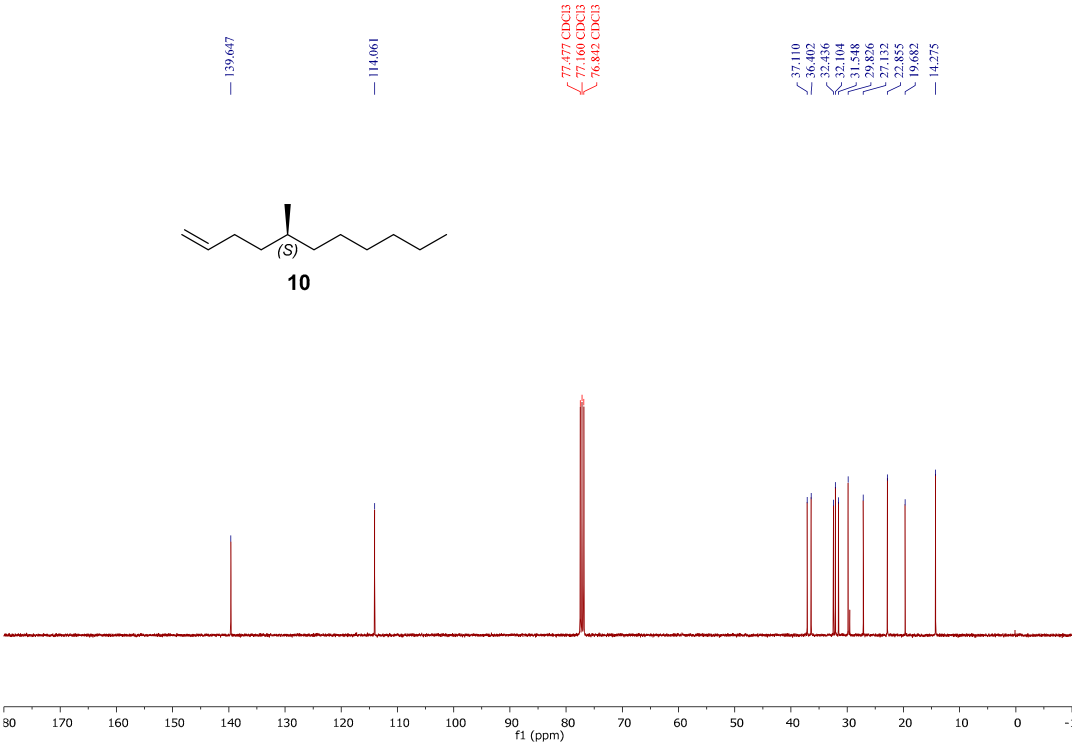

Figure S9. ^{1}H NMR Spectrum of compound **11** (400 MHz, $CDCl_3$)

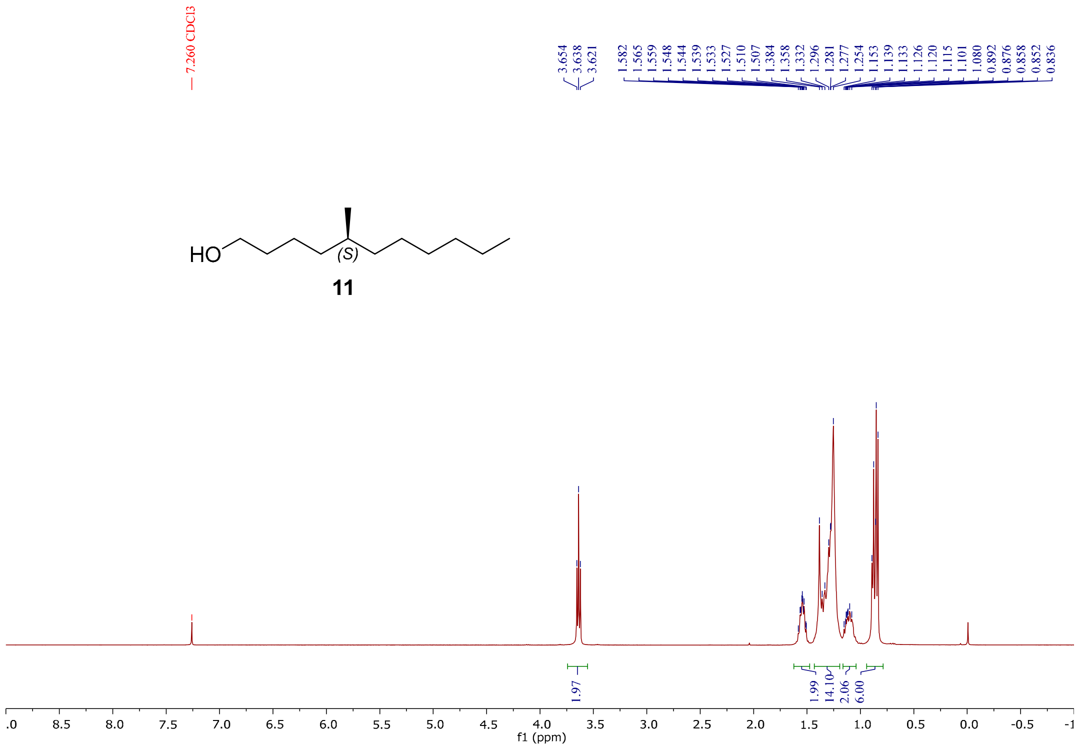

Figure S10. ^{13}C NMR Spectrum of compound **11** (101 MHz, $CDCl_3$)

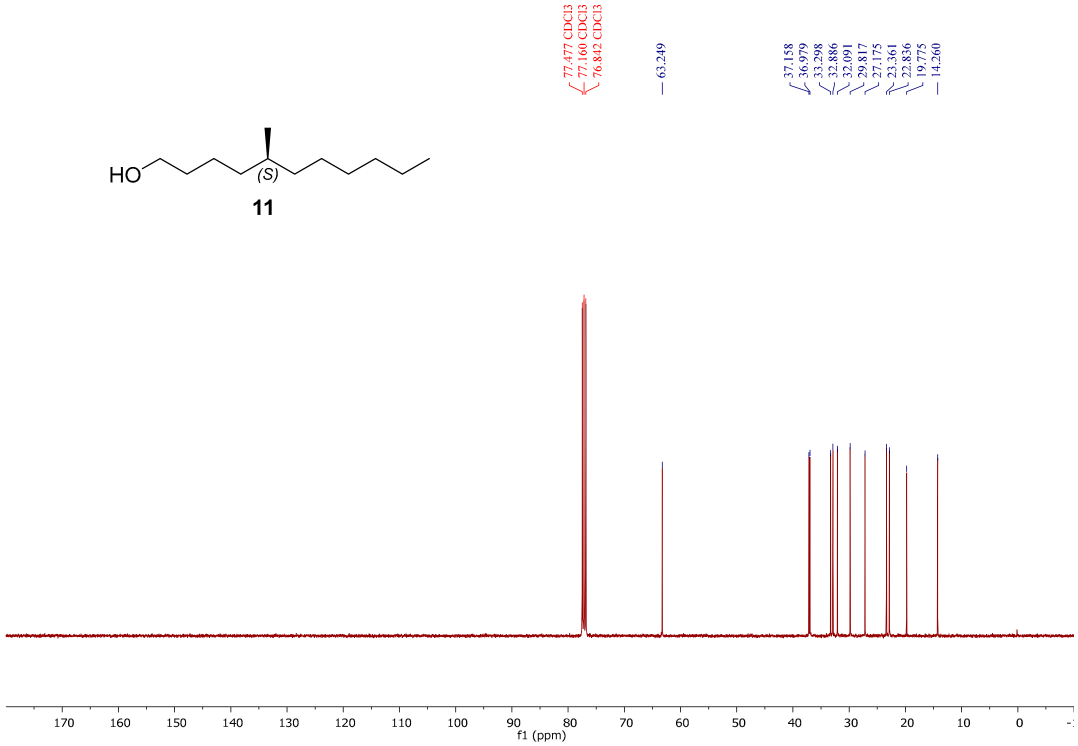

Figure S11. ^{1}H NMR Spectrum of compound **12** (500 MHz, $CDCl_3$)

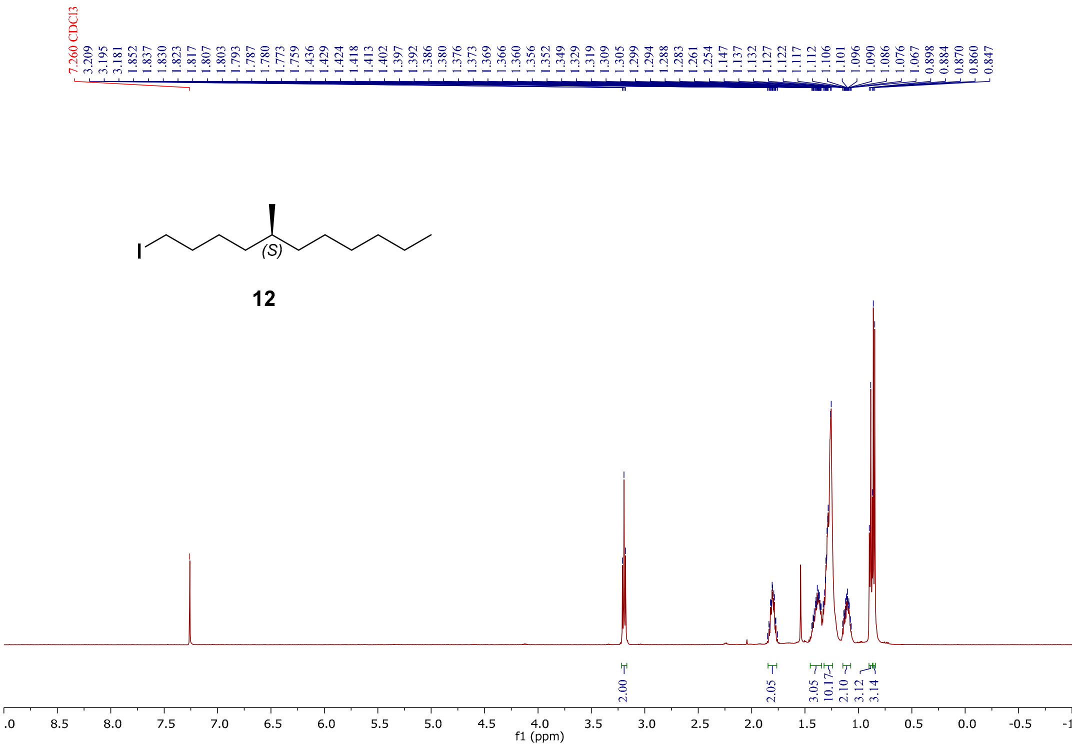

Figure S12. ^{13}C NMR Spectrum of compound **12** (126 MHz, $CDCl_3$)

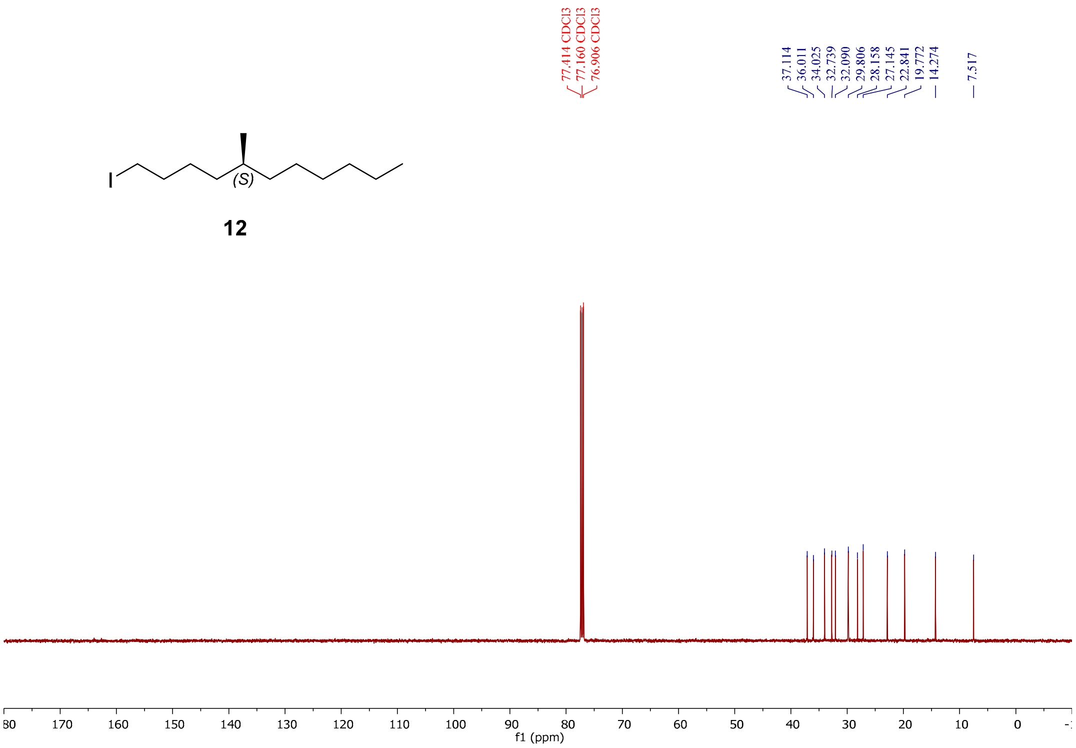

Figure S13. ^{1}H NMR Spectrum of compound **13** (500 MHz, $CDCl_3$)

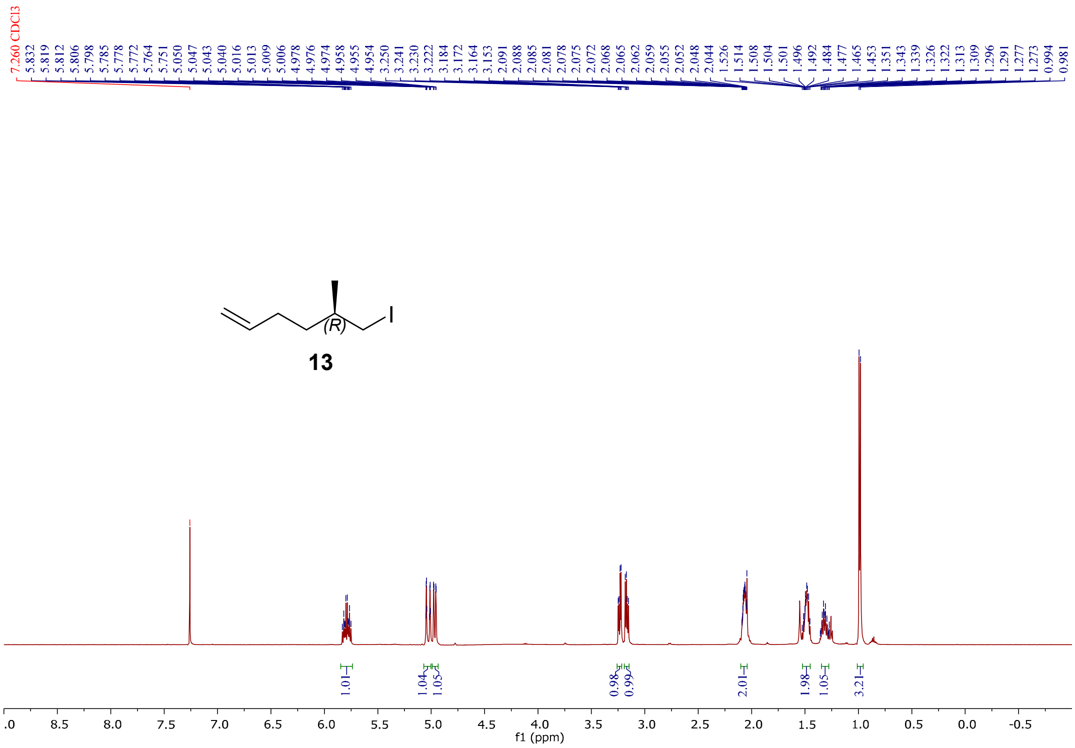

Figure S14. ^{13}C NMR Spectrum of compound **13** (126 MHz, $CDCl_3$)

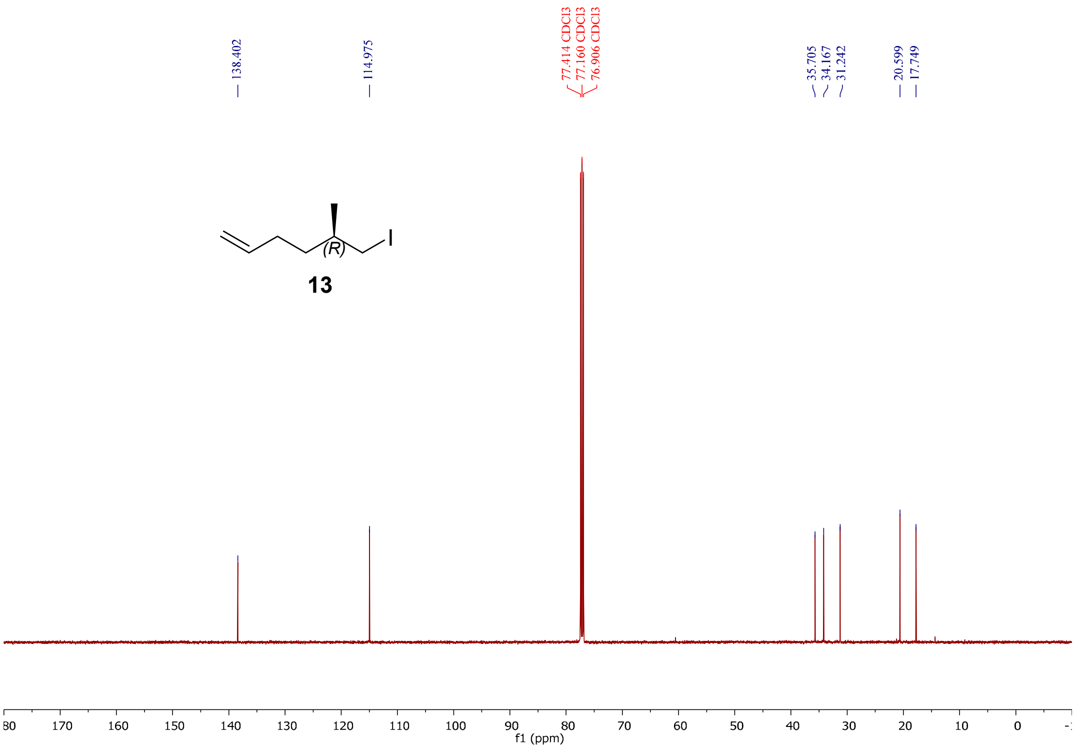

Figure S15. ^{1}H NMR Spectrum of compound **13** (500 MHz, $CDCl_3$)

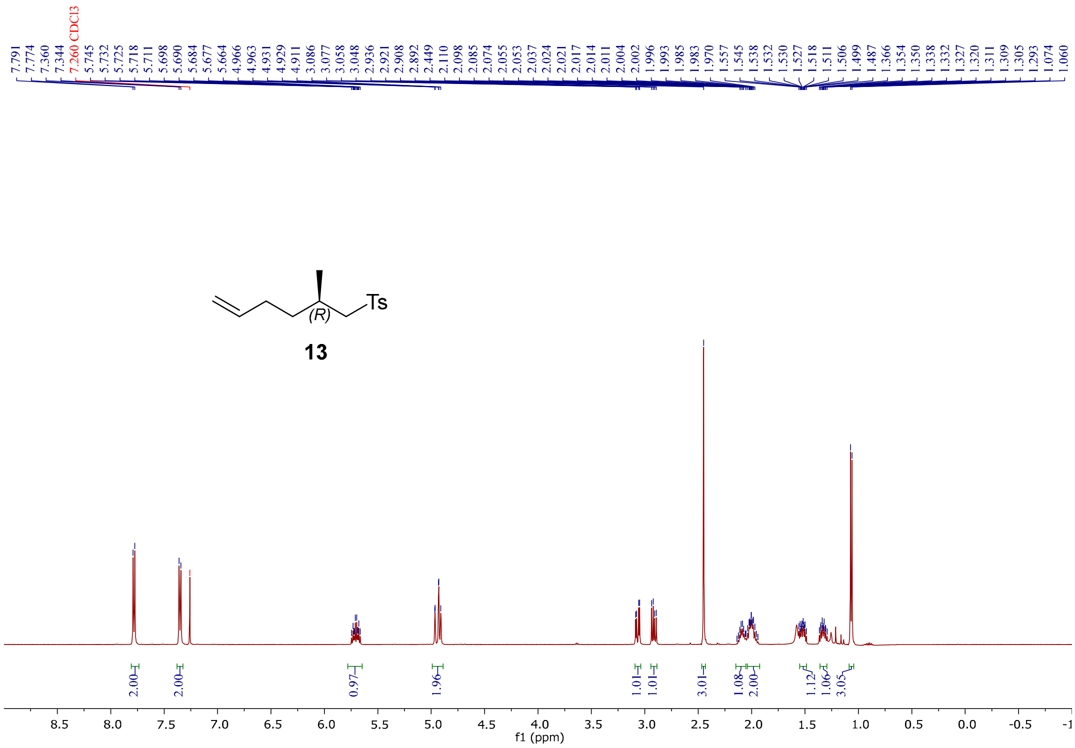

Figure S16. ^{13}C NMR Spectrum of compound **13** (126 MHz, $CDCl_3$)

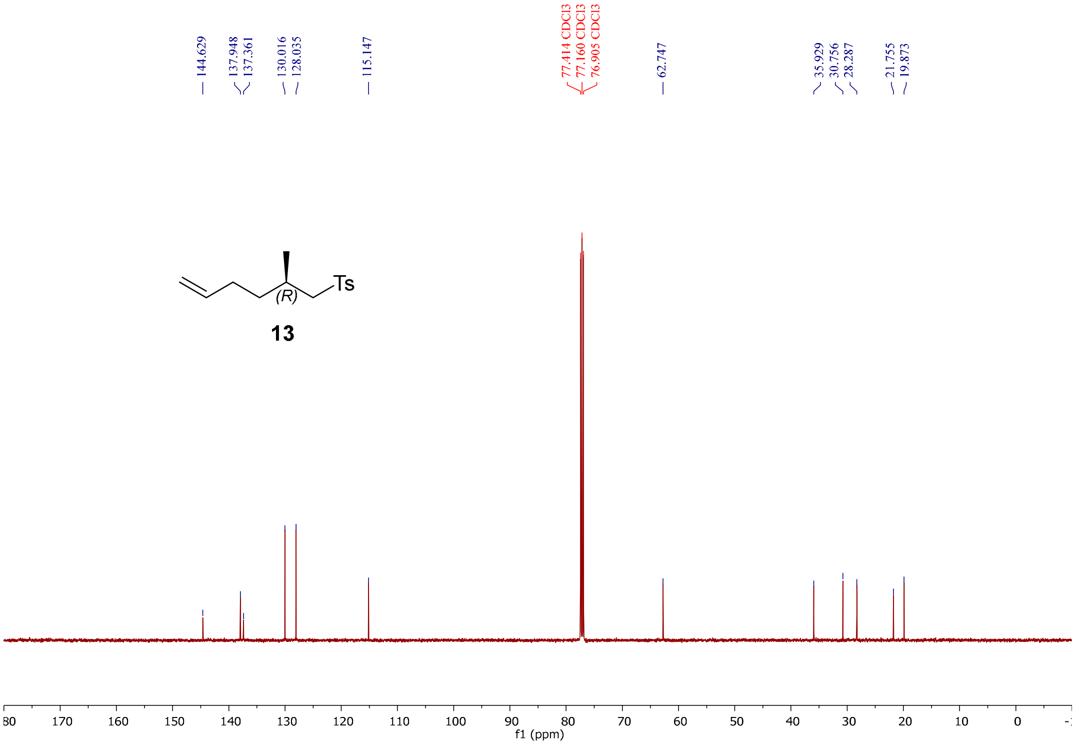

Figure S17. ^{1}H NMR Spectrum of compound **15** (500 MHz, $CDCl_3$)

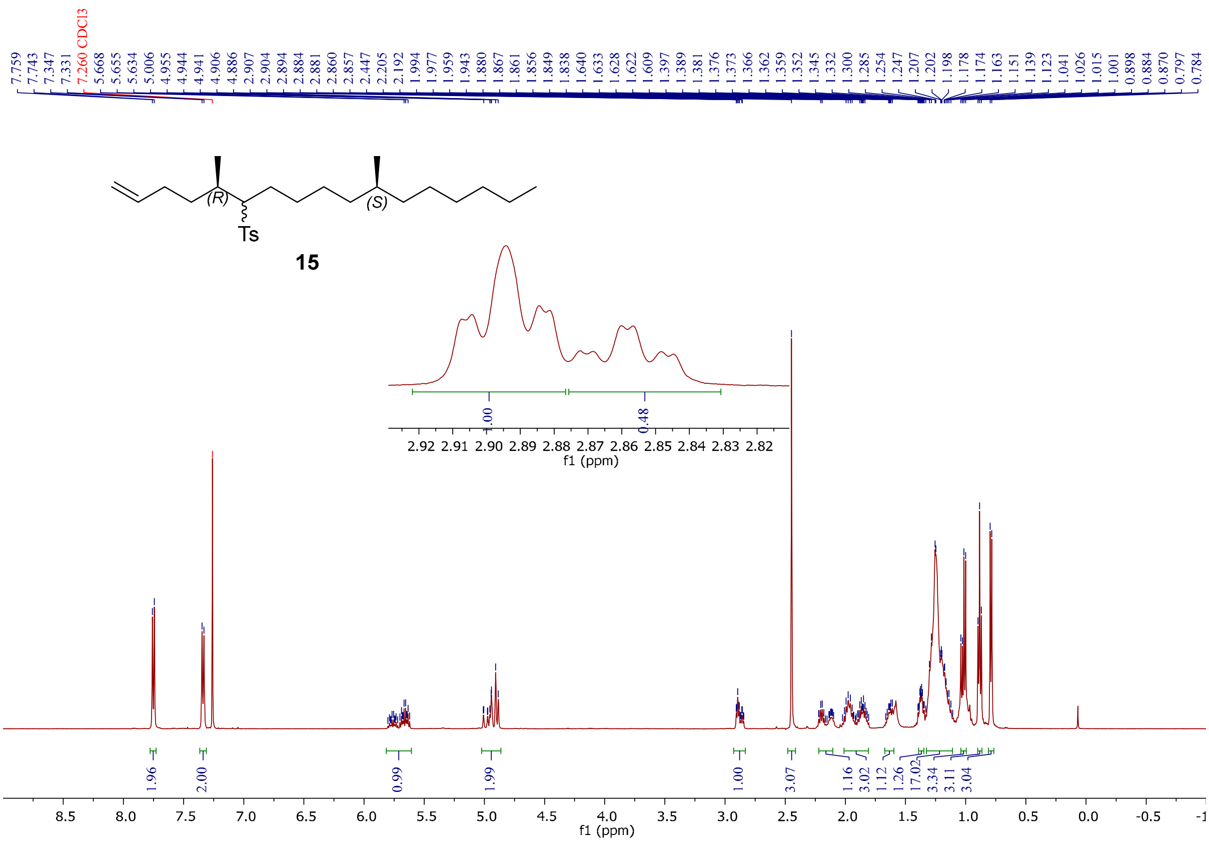

Figure S18. ^{13}C NMR Spectrum of compound **15** (126 MHz, $CDCl_3$)

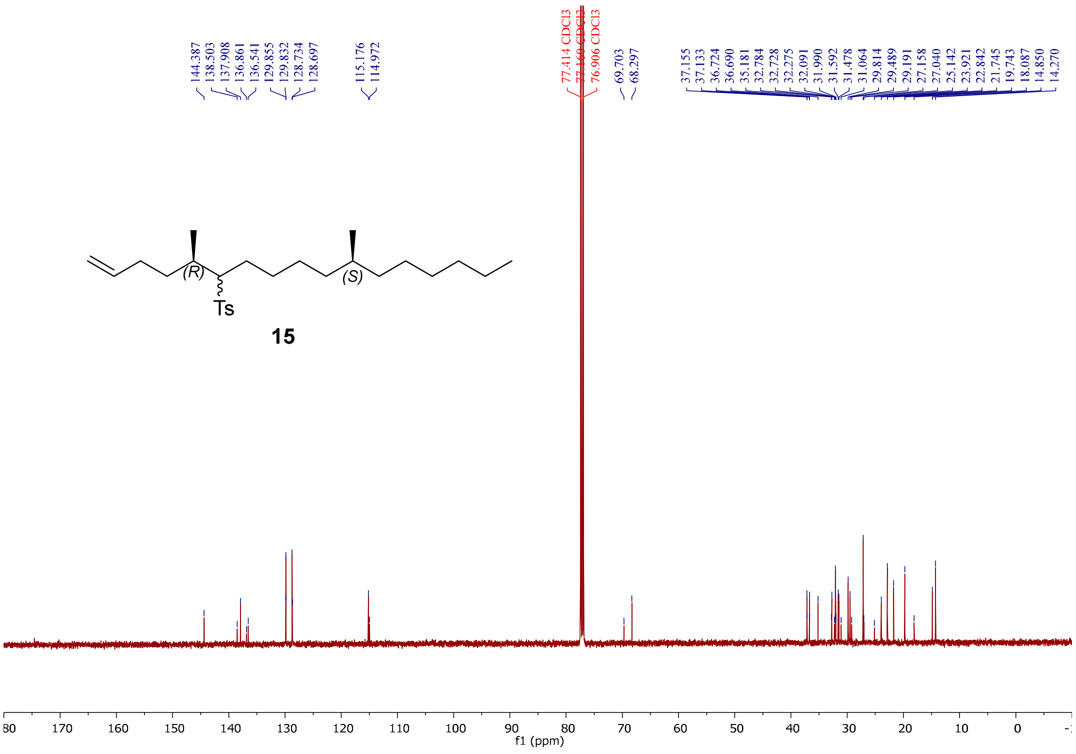

Figure S19. ^{1}H NMR Spectrum of compound **1** (500 MHz, $CDCl_3$)

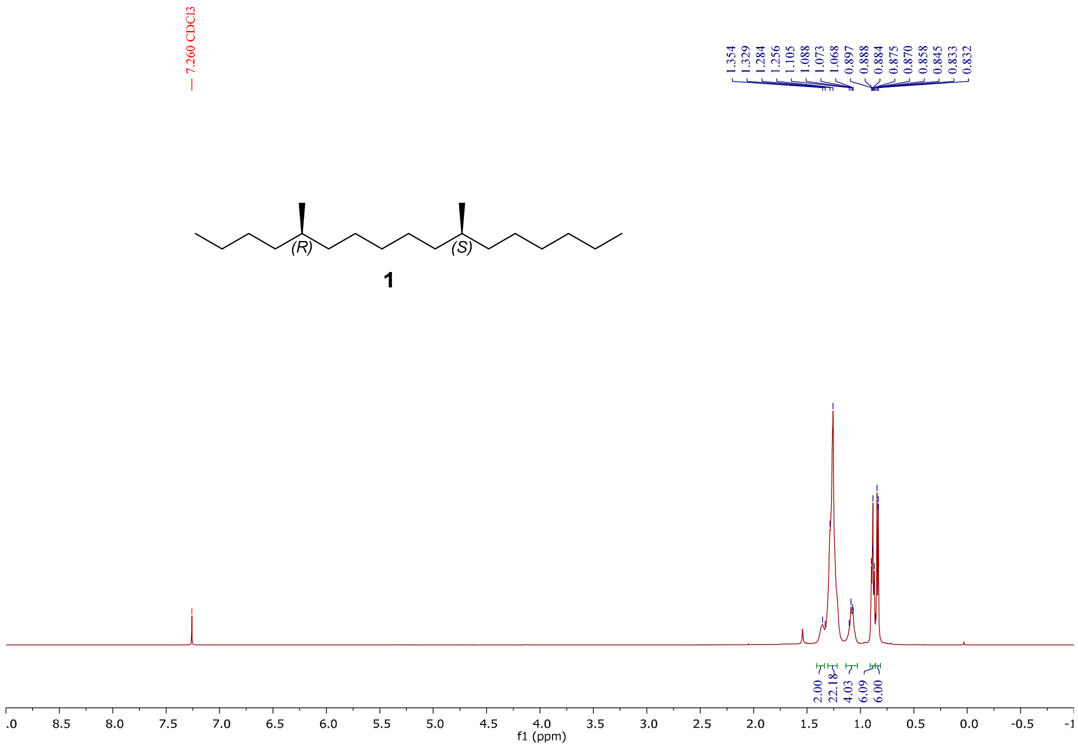

Figure S20. ^{13}C NMR Spectrum of compound **1** (126 MHz, $CDCl_3$)

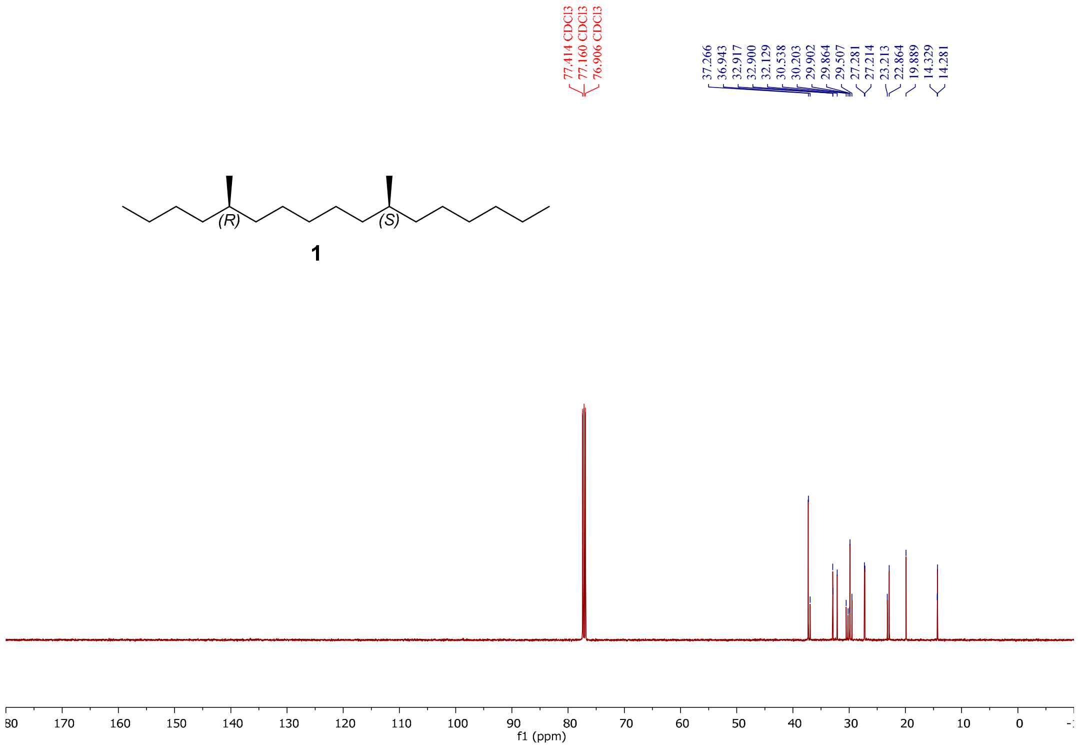

Figure S21. ^{1}H NMR Spectrum of compound **2** (400 MHz, $CDCl_3$)

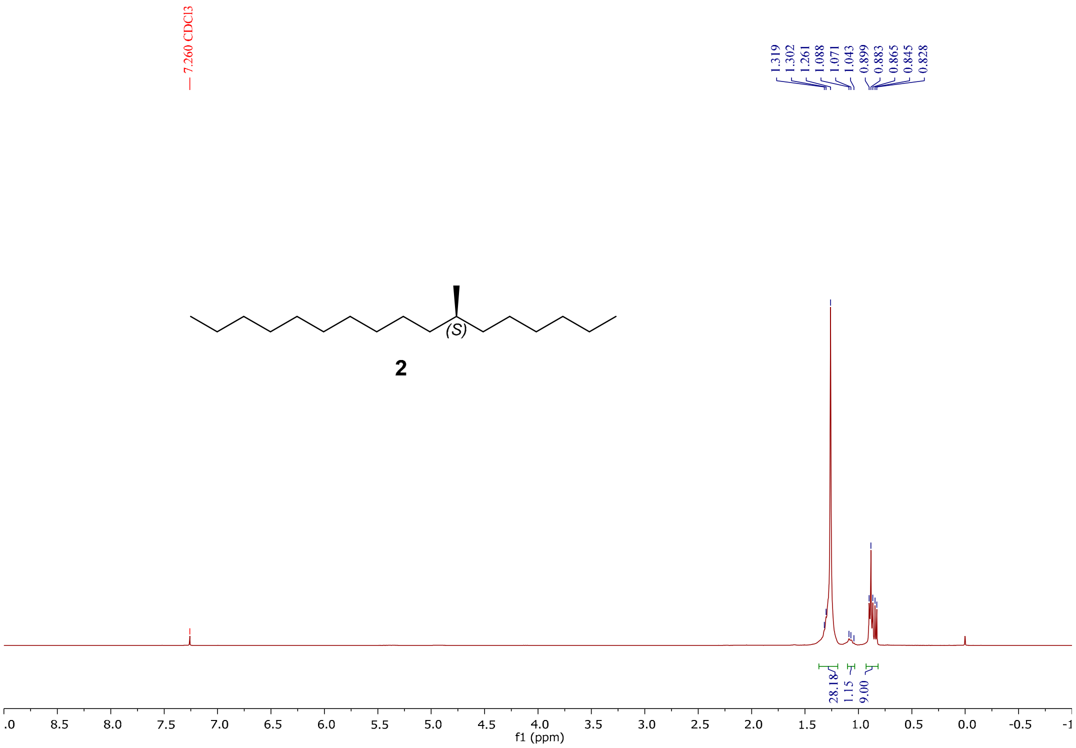

Figure S22. ^{13}C NMR Spectrum of compound **2** (101 MHz, $CDCl_3$)

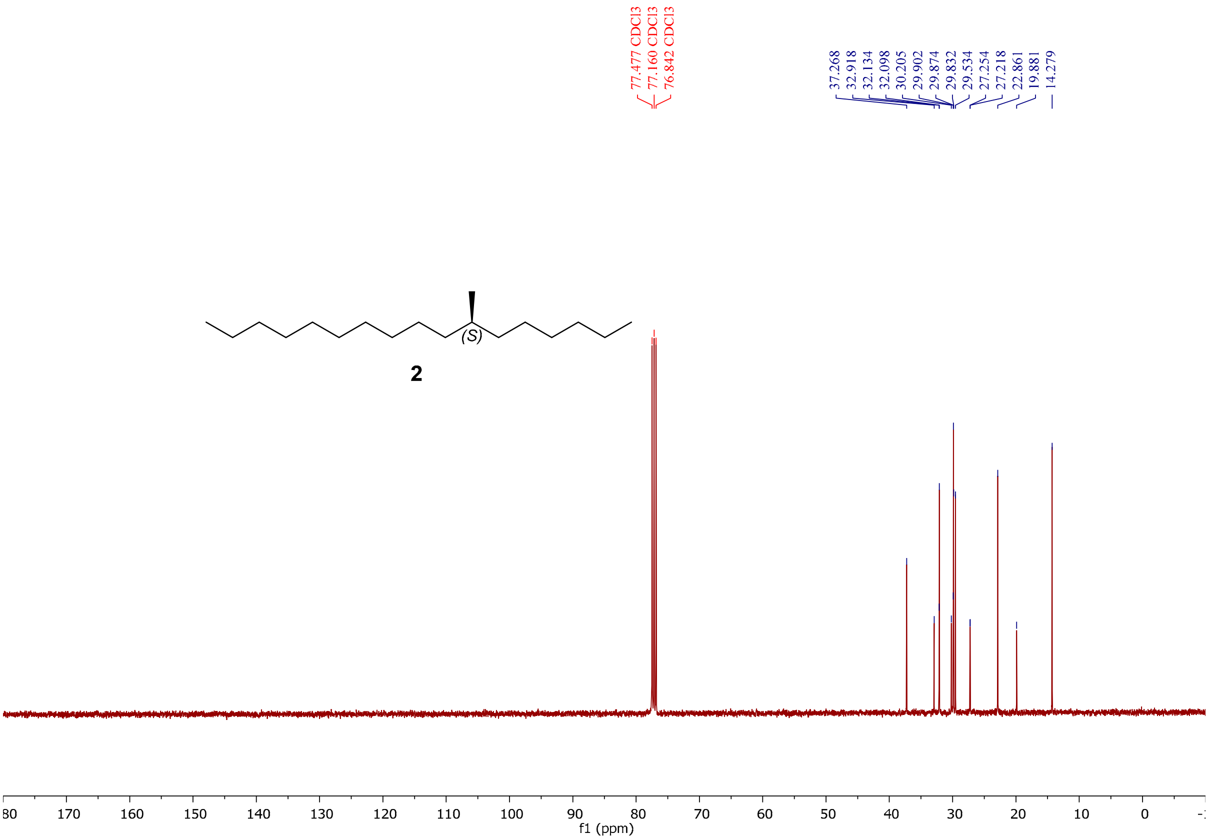

Figure S23. ^{1}H NMR Spectrum of compound **19** (500 MHz, $CDCl_3$)

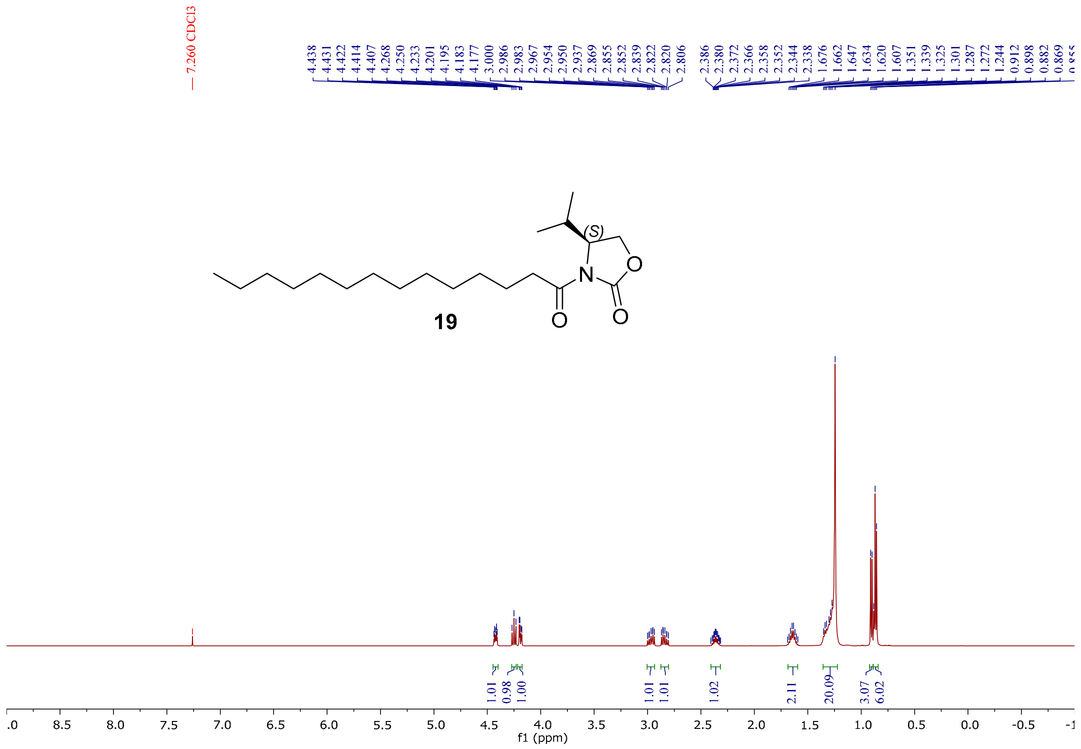

.

Figure S24. ^{13}C NMR Spectrum of compound **19** (126 MHz, $CDCl_3$)

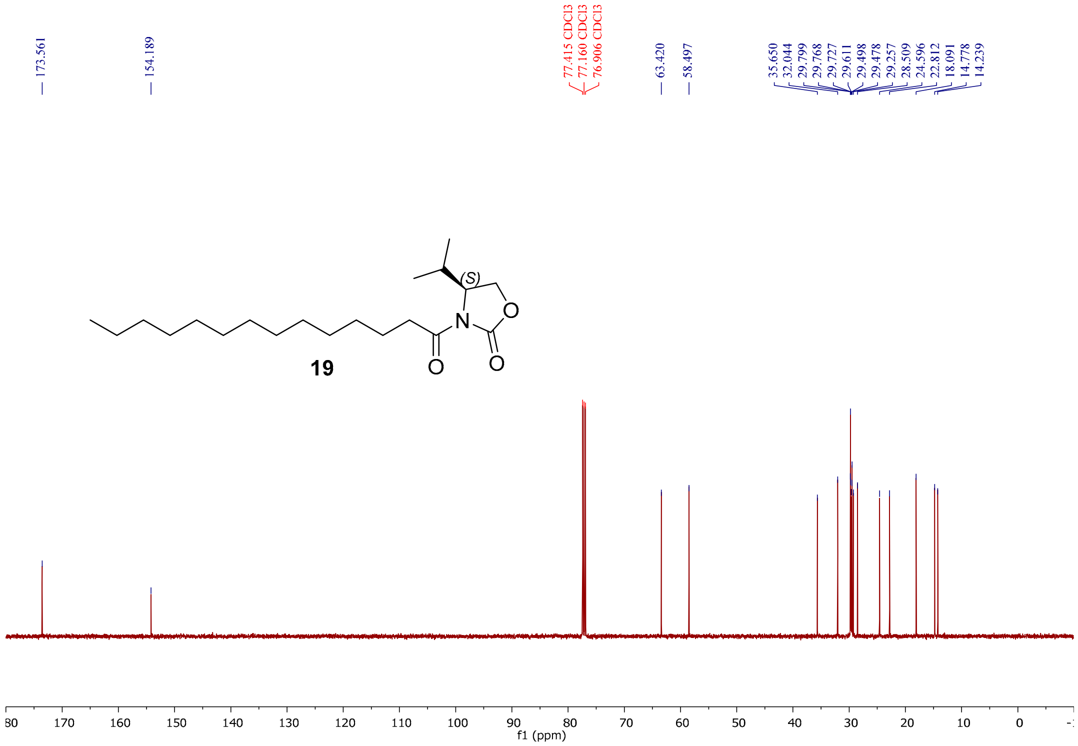

Figure S25. ^{1}H NMR Spectrum of compound **20** (500 MHz, $CDCl_3$)

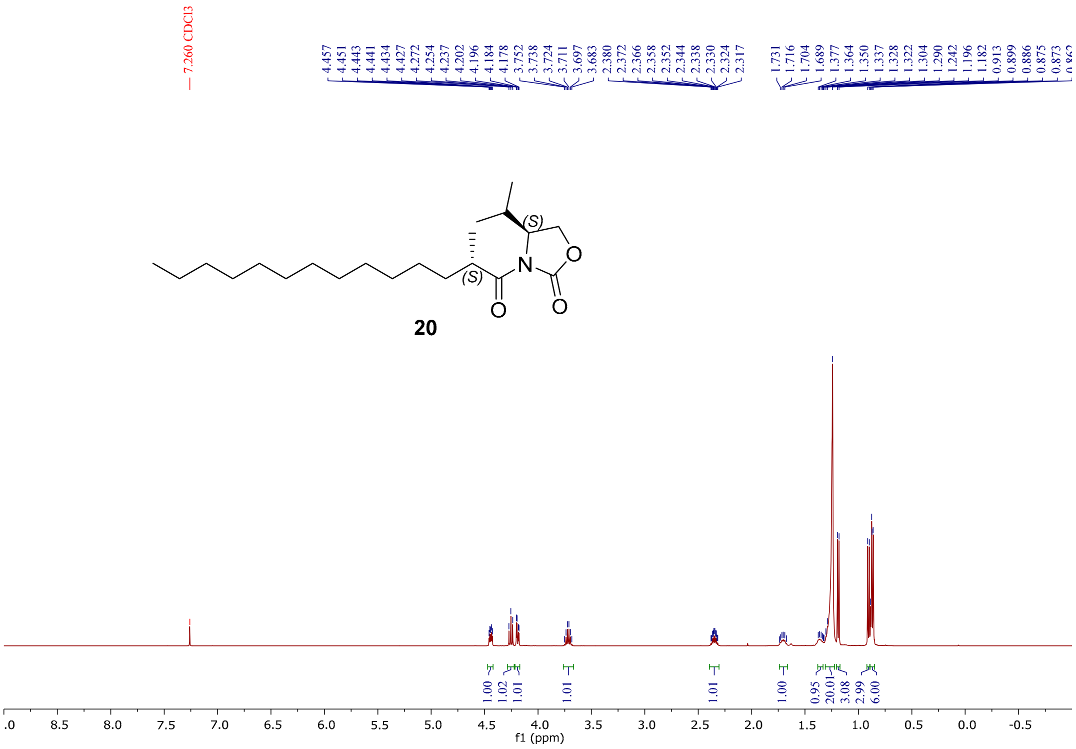

Figure S26. ^{13}C NMR Spectrum of compound **20** (126 MHz, $CDCl_3$)

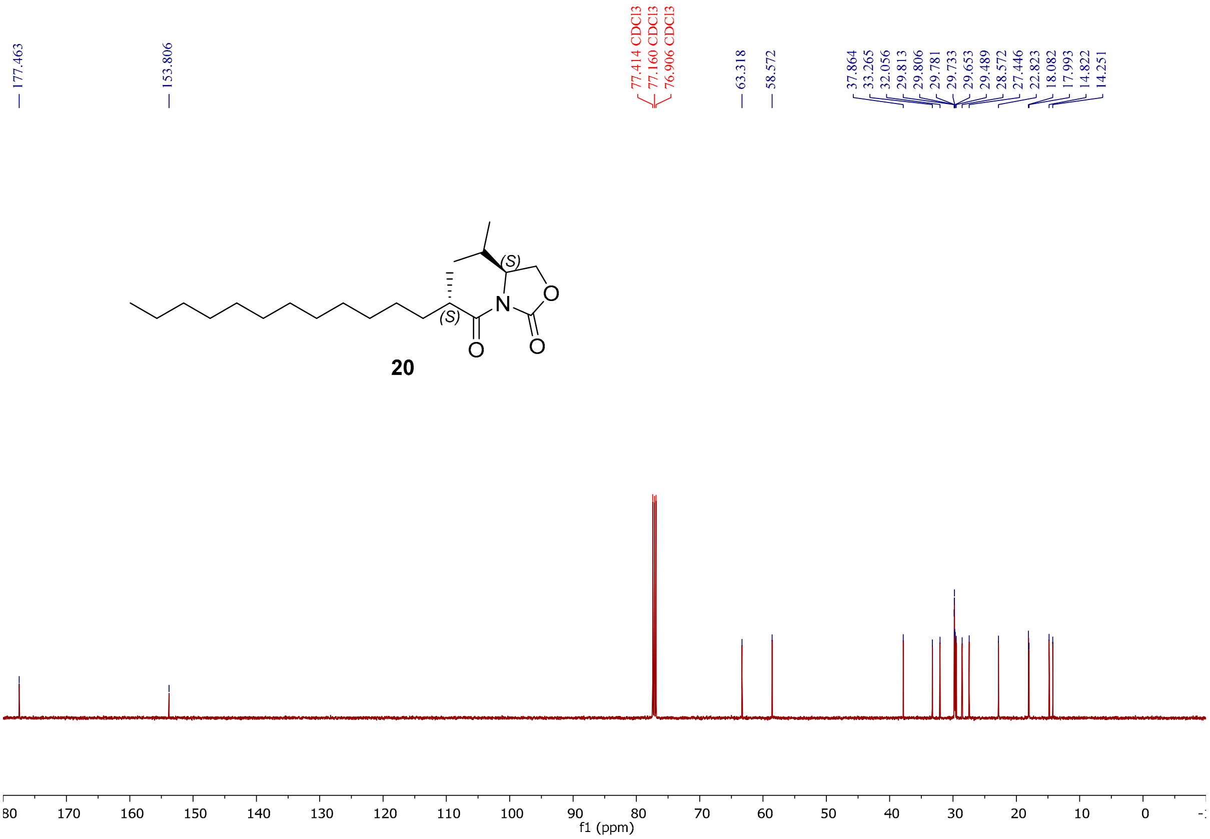

Figure S27. ^{1}H NMR Spectrum of compound **21** (500 MHz, $CDCl_3$)

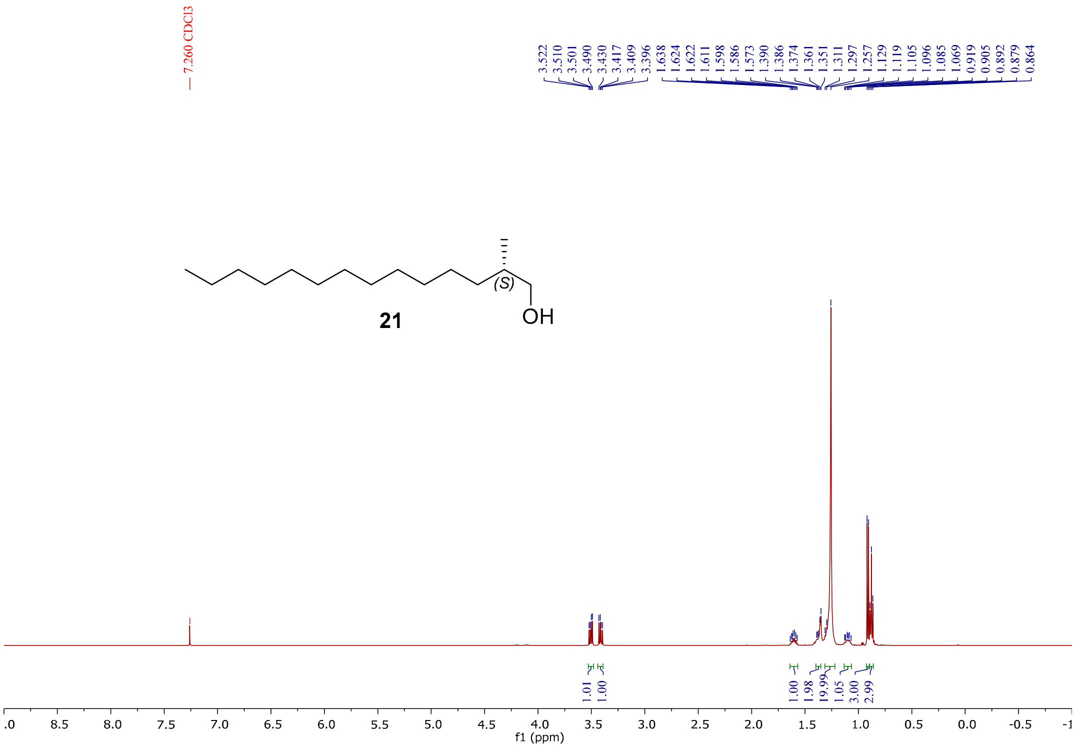

Figure S28. ^{13}C NMR Spectrum of compound **21** (126 MHz, $CDCl_3$)

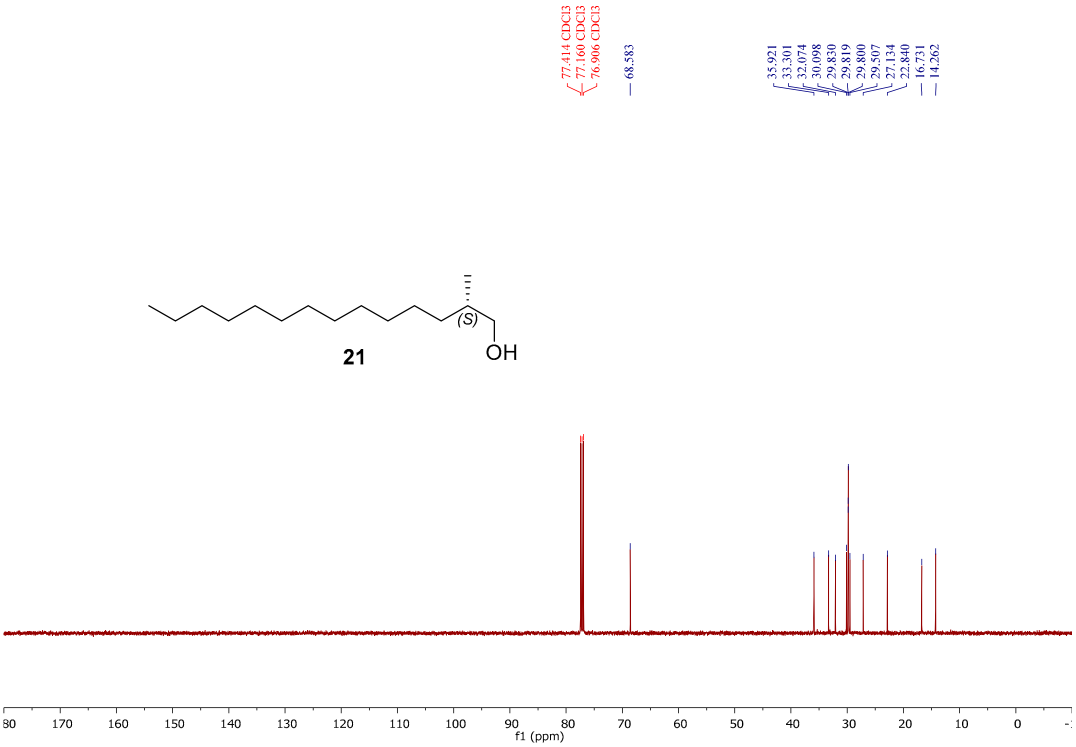

Figure S29. ^{1}H NMR Spectrum of compound **3** (400 MHz, $CDCl_3$)

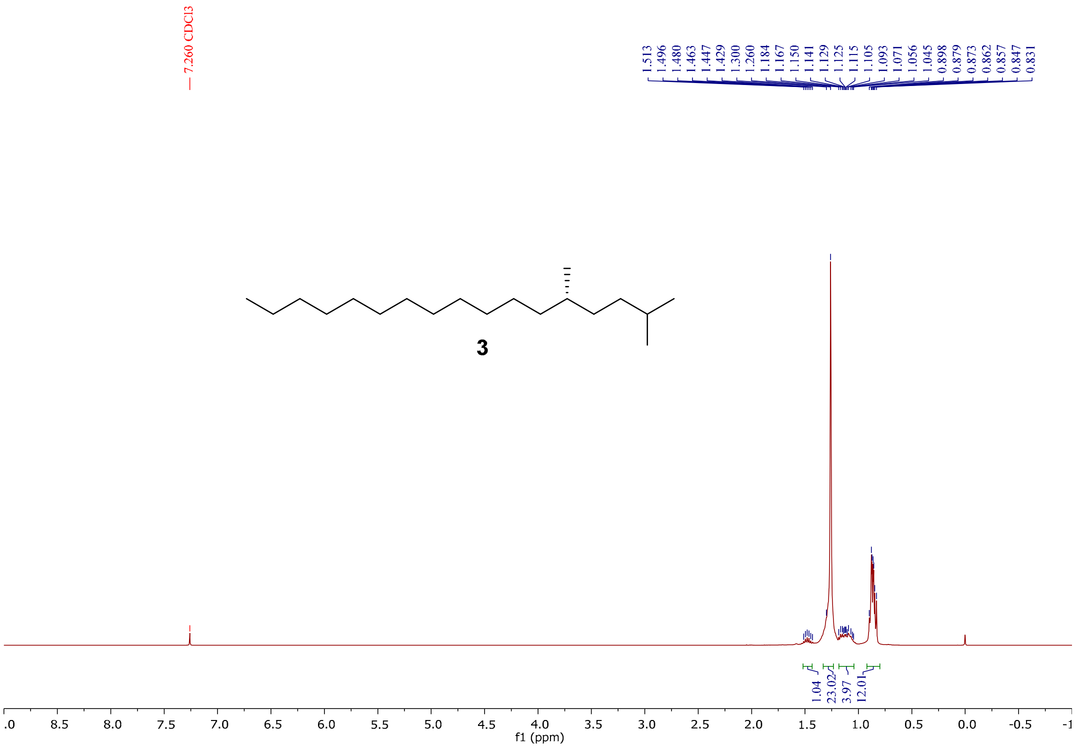

Figure S30. ^{13}C NMR Spectrum of compound **3** (101 MHz, $CDCl_3$)

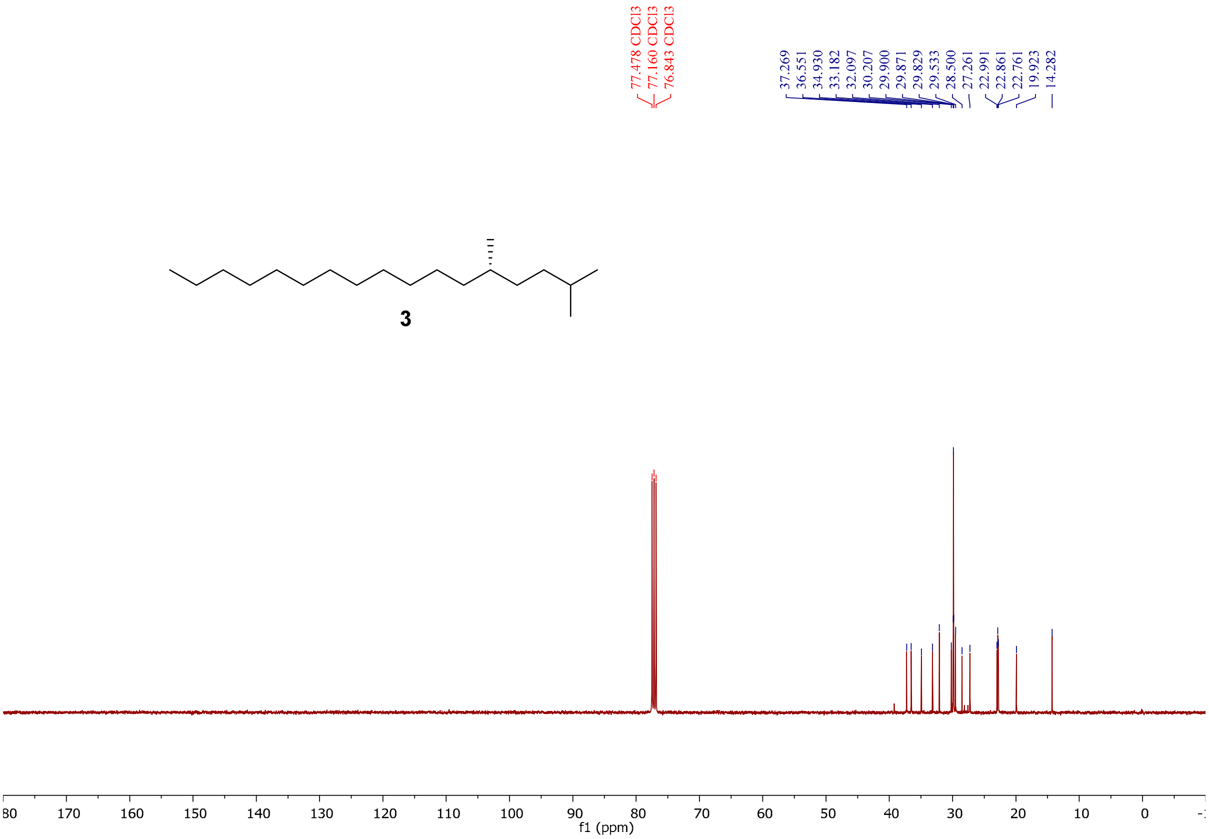

Figure S31. ^{1}H NMR Spectrum of compound **23** (400 MHz, $CDCl_3$)

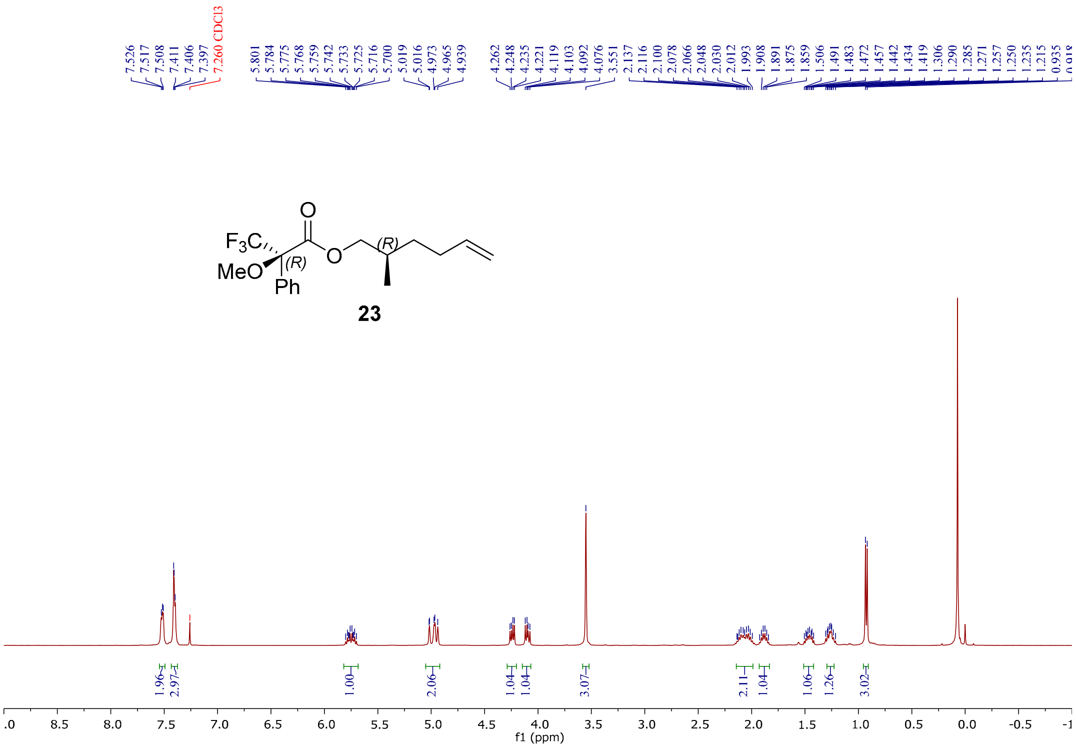

Figure S32. ^{1}H NMR Spectrum of compound **24** (400 MHz, $CDCl_3$)

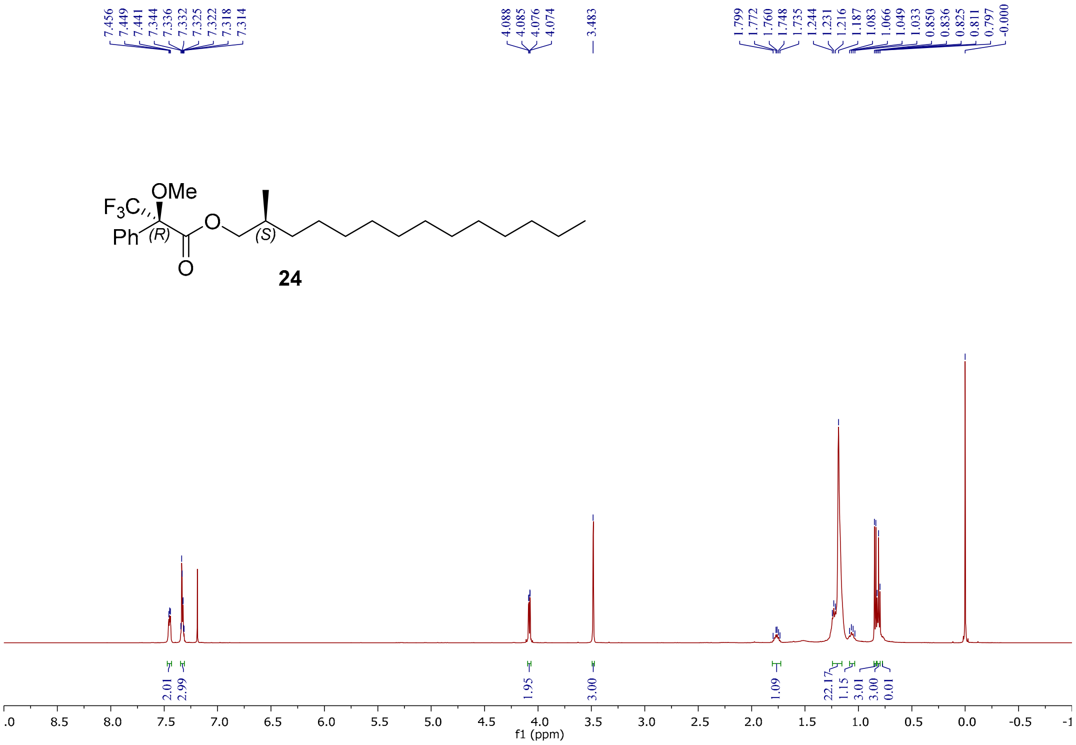

4. Y-tube and cage experimental data

Table S1. Effect of pheromone components **1**, **2**, and **3** on the attraction of male *Semiothisa cinerearia* (Bremer & Grey) to the Y-shaped tube

Entry	compound	Tested insects	Chose treatment arm	Chose control arm (*n*-Hexane)	No choice
1	1（0.1mg）	50	26	20	4
2	1（0.1mg）	50	27	18	5
3	1（0.1mg）	50	26	22	2
4	2（0.1mg）	50	20	27	3
5	2（0.1mg）	50	23	22	5
6	2（0.1mg）	50	21	25	4
7	3（0.1mg）	50	21	26	3
8	3（0.1mg）	50	24	20	6
9	3（0.1mg）	50	22	26	2

Table S2. Experiment on attracting male *Semiothisa cinerearia* (Bremer & Grey) to Y-shaped tube by different doses of pheromone component **1**

Entry	Dose	Tested insects	Chose treatment arm	Chose control arm (*n*-Hexane)	No choice
1	1μg	50	24	22	4
2	1μg	50	21	25	4
3	1μg	50	24	24	2
4	10μg	50	25	23	2
5	10μg	50	24	22	4
6	10μg	50	26	21	3
7	100μg	50	27	19	4
8	100μg	50	27	21	2
9	100μg	50	25	20	5
10	1000μg	50	29	18	3
11	1000μg	50	25	19	6
12	1000μg	50	27	18	5

Table S3. Cage experiment on attracting male *Semiothisa cinerearia* (Bremer & Grey) with different doses of pheromone component **1**

Entry	Dose	Tested insects	Chose treatment arm	Chose control arm (*n*-Hexane)
1	1μg	20	11	9
2	1μg	20	11	9
3	1μg	20	9	11
4	10μg	20	11	9
5	10μg	20	12	8
6	10μg	20	14	6
7	100μg	20	12	8

8	100μg	20	13	7
9	100μg	20	14	6
10	1000μg	20	13	7
11	1000μg	20	14	6
12	1000μg	20	12	8

5. Reference

1. Yu, S.; Yuan, G.; Liu, J.; Bian, Q.; Wang, M.; Zhong, J.C. (2023). Asymmetric synthesis of the sex pheromone of the apple leafminer, *Lyonetia prunifoliella*. *Chirality*, *35*, 118-128.
